# Cell type phylogenetics informs the evolutionary origin of echinoderm larval skeletogenic cell identity

**DOI:** 10.1038/s42003-019-0417-3

**Published:** 2019-05-03

**Authors:** Eric M. Erkenbrack, Jeffrey R. Thompson

**Affiliations:** 1 Department of Ecology and Evolutionary Biology, Yale University, New Haven, CT 06511 USA; 2 Yale Systems Biology Institute, Yale University, West Haven, CT 06516 USA; 3 Department of Geosciences, Baylor University, Waco, TX 76706 USA; 4 Department of Earth Sciences, University of Southern California, Los Angeles, CA 90089-0740 USA

**Keywords:** Phylogenetics, Evolutionary developmental biology

## Abstract

The multiplicity of cell types comprising multicellular organisms begs the question as to how cell type identities evolve over time. Cell type phylogenetics informs this question by comparing gene expression of homologous cell types in distantly related taxa. We employ this approach to inform the identity of larval skeletogenic cells of echinoderms, a clade for which there are phylogenetically diverse datasets of spatial gene expression patterns. We determined ancestral spatial expression patterns of *alx1, ets1, tbr, erg*, and *vegfr*, key components of the skeletogenic gene regulatory network driving identity of the larval skeletogenic cell. Here we show ancestral state reconstructions of spatial gene expression of extant eleutherozoan echinoderms support homology and common ancestry of echinoderm larval skeletogenic cells. We propose larval skeletogenic cells arose in the stem lineage of eleutherozoans during a cell type duplication event that heterochronically activated adult skeletogenic cells in a topographically distinct tissue in early development.

## Abstract (web summary)

Eric Erkenbrack and Jeffrey Thompson have performed ancestral state reconstructions of echinoderm larval skeletogenic cells to examine their relatedness and provide insights into cell-type evolution. They propose that larval skeletogenic cells originated in the stem lineage of eleutherozoans.

## Introduction

Cell types are evolutionary units that have diversified in structure and function since the dawn of multicellularity^1^. During development, generational iterations of cell types are established by gene regulatory networks (GRNs) comprised of regulatory molecules, e.g., transcription factors and signaling pathways, interacting with cell type-specific DNA regulatory elements^2^ to control gene expression. Similarly, in evolution, cell types form lineages of common ancestry that are maintained over vast expanses of evolutionary time by networks of coding and noncoding regulatory interactions called cell type identity networks^1,3^. The diversity of functionally distinct cells in multicellular organisms suggests that their underlying identities are labile enough to generate novelty but also rigid enough to buffer change. Understanding how cell type identity evolves is thus key to explaining cell type diversity. It has been proposed that novel cell types are generated by cell type splitting events that give rise to sister cell types from ancestral cell types^4^, a model which has been advanced to explain the origins of vertebrate ciliary photoreceptor cells^4^ and mammalian endometrial stromal fibroblasts^5,6^. A putative mechanism of cell type splitting is cell type duplication, during which sister cell types arise within different developmental lineages or developmental contexts. Whereas it is clear how these proposed mechanisms would contribute to cell type diversity, detailed examples are still uncommon in the literature.

Cell-type phylogenetics and phylogenetic comparative methods have the potential to resolve questions regarding the evolutionary origin of cell types, and the evolution of the larval skeletogenic cells of echinoderms offers one such opportunity. These cells are specified by an extensively studied GRN^7–20^ and occur in indirect developing larvae of three of the five classes of echinoderms: ophiuroids (brittle stars), holothurians (sea cucumbers), and echinoids (sea urchins). In echinoids and ophiuroids, larval skeletogenic cells synthesize an elaborate larval skeleton that aids in structural support, feeding, and locomotion^21^. The evolutionary relatedness of these cells and the homology of larval skeletons has long been a point of contention, with various arguments posited for^22–24^ or against^25–31^ homology. As all fossil and extant echinoderms possess adult skeletogenic cells and only a subset of echinoderm lineages are known to possess larval skeletogenic cells^32,33^, there is agreement that adult skeletogenic cells evolved first. One hypothesis is that heterochronic activation of the adult skeletogenic GRN during early development underlay the origin of the larval skeletogenic cell^33^. It is still unclear where and how many instances of this heterochronic activation occurred in the echinoderm phylogeny. As the most recent phylogeny of echinoderms places asterozoans (asteroids+ophiuroids) as a sister clade to echinozoans (echinoids+holothurians), it is widely thought that the elaborate larval skeletons in echinoids and ophiuroids are the result of independent evolutionary events^13,19,29,34,35^. Importantly, only recently have developmental gene expression data for holothurians come to light^20^. These sea cucumbers also possess larval spicules, however, they do not elaborate into a larval skeleton^24^, suggesting that their inclusion may help  clarify the evolutionary relatedness of larval skeletogenic cells.

Here, we frame spatial gene expression data of regulatory genes driving euechinoid larval skeletogenic cell identity from numerous echinoderms in the context of cell type evolution to inform the relatedness of echinoderm larval skeletogenic cells. We collated spatial gene expression patterns for regulatory genes that are important in specification of euechinoid larval skeletogenic cells. We utilized ancestral state reconstruction to estimate the probability of extant spatial gene expression patterns at each node of the eleutherozoan echinoderm phylogeny. Our analyses are consistent with the hypothesis that larval skeletogenic cells arose once in the stem lineage of eleutherozoan echinoderms. We propose that this event was a cell-type duplication event involving activation of the adult skeletogenic cell during early development. This evolutionary event gave rise to a sister cell type, the larval skeletogenic cell, that was subsequently individuated or lost in different lineages of extant eleutherozoans. Our analysis affords a method to rigorously determine ancestral states of spatial gene expression patterns, thereby revealing how cell-type identity changes over vast expanses of evolutionary time.

## Results

### Gene selection and ancestral state reconstruction

**Fig. 1:**
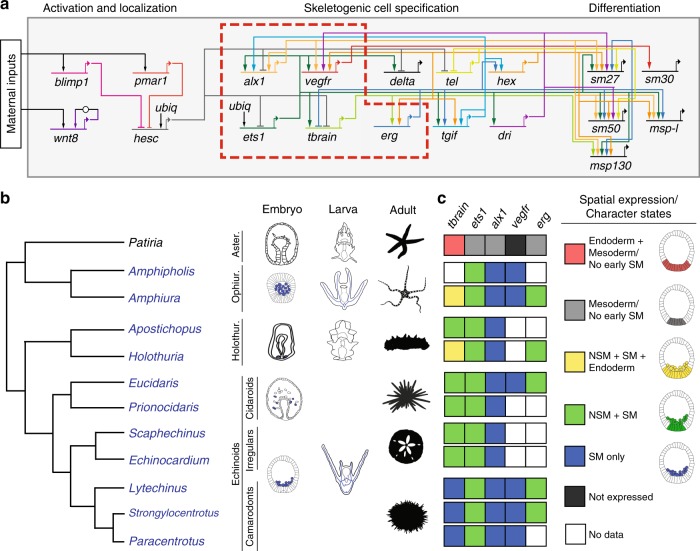
Gene regulatory network of the larval skeletogenic cell and spatial distribution of skeletogenic regulatory genes early in development of eleutherozoan echinoderms studied herein. **a** Gene regulatory network of the *S. purpuratus* larval skeletogenic cell type showing regulatory genes and their interactions. This study focuses on the evolution of the genes shown in the red dashed box, *viz. alx1, erg, ets1, tbrain*, and *vegfr*. The network is based on the GRN found at http://echinobase.org/endomes and various studies^11,35^. **b** Phylogeny of eleutherozoan echinoderms showing classes, typical indirect embryonic and larval developmental stages with skeletogenic cells and/or larval skeleton in blue, and typical adult forms. Aster, Asteroidea; Ophiur, Ophiuroidea; Holothur, Holothuroidea. Taxa in blue indicate presence of larval skeletogenic cells. **c** Spatial distribution of four transcription factors shown to be important for specification of euechinoid larval skeletogenic cells in early development of eleutherozoan echinoderms. Character states used for ancestral state reconstruction are shown at right along with examples of how states were scored. SM skeletogenic mesenchyme, NSM nonskeletogenic mesenchyme. Silhouette images in **b** were created individually or are in the public domain with the following exceptions: ophiuroid (credit Noah Shlottman, photo from Casey Dunn), clypeasteroid (credit Michelle Site), and camarodont echinoid (credit Frank Förster based on a picture by Jerry Kirkhart; modified by T. Michael Keesey), all of which are used under license http://creativecommons.org/licenses/by-sa/3.0/. No changes were made to the images

**Fig. 2:**
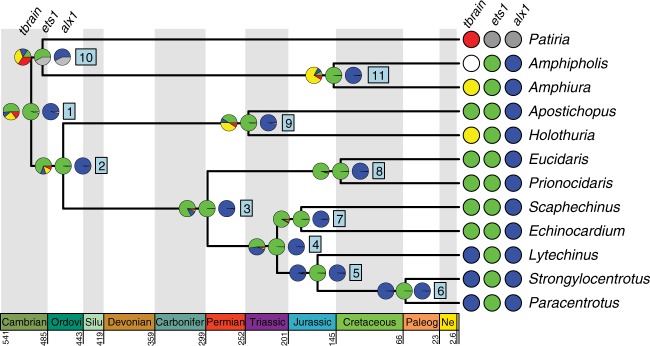
Ancestral state reconstruction of spatial gene expression data using a single-rate Markov model^42^ across eleutherozoan echinoderms. Ancestral states of *alx1, ets1*, and *tbrain*, three transcription factors critical to euechinoid larval skeletogenic cell specification. Geological timescale shown at bottom. Nodes are represented by boxed numbers. For each extant character state, the probability that it occurs at the ancestral node was estimated. The pie charts at each node show the mean posterior probability for each spatial expression pattern of the genes shown at right. The colors represent the character states shown in Fig. 1c. Abbreviations in the geological timescale are as follows: Ordovi Ordovician, Silur Silurian, Carbonifer Carboniferous, Paleog Paleogene, Ne Neogene

**Fig. 3:**
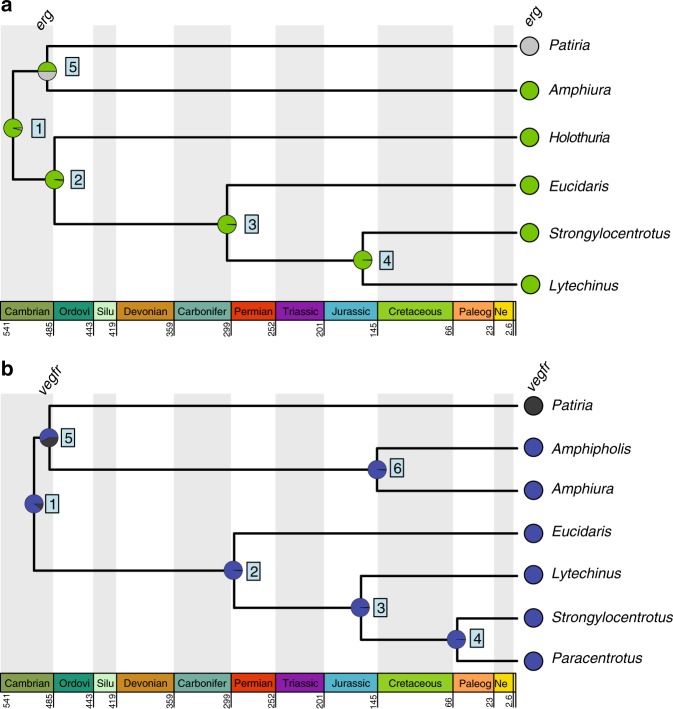
Ancestral state reconstruction of spatial gene expression data for *erg* and *vegfr* using a single-rate Markov model^42^ across eleutherozoan echinoderms. **a** Ancestral state reconstruction of spatial expression patterns of the transcription factor *erg*. **b** Ancestral state reconstruction of spatial expression patterns of the signaling gene *vegfr*. Geological timescale shown at bottom. Nodes are represented by boxed numbers. For each extant character state, the probability that it occurs at the ancestral node was estimated. The pie charts at each node show the mean posterior probability for each spatial expression pattern of the genes shown at right. The colors represent the character states shown in Fig. 1c. Abbreviations in the geological timescale are as follows: Ordovi Ordovician, Silur Silurian, Carbonifer Carboniferous, Paleog Paleogene, Ne Neogene

We conducted ancestral state reconstruction on spatial expression patterns from early larval stages and on regulatory genes for which there exist data in at least five taxa in widely diverged clades (see Methods). Experimental studies have revealed transcription factors, e.g., *alx1, erg, ets1*, and *tbrain*, and signaling pathways, e.g., VEGF signaling, that are critical for larval skeletogenic cell specification^35^. The granular molecular detail of this process in the model euechinoid sea urchin *Strongylocentrotus purpuratus* has motivated comparative evolutionary developmental research in numerous phylogenetically distant echinoderms^36–39^. We assembled a database of spatial gene-expression data for similar timepoints in early development for *alx1, erg, ets1, tbrain*, and *vegfr*, regulatory genes that underlie specification of these cells based on the published GRN at http://echinobase.org/endomes/, and scored spatial distribution of their expression as character states (Fig. 1 and Supplementary Data 1). We used phylogenetic comparative methods^40–44^ to reconstruct the ancestral spatial expression patterns of regulatory genes in eleutherozoan echinoderms. To explicitly frame spatial gene expression data in the context of echinoderm phylogeny, we included all species that have both spatial gene-expression data for regulatory genes critical to skeletogenic cell specification as well as gene sequences available for divergence time estimation (see Methods). We used a backbone tree from a composite of published echinoderm molecular phylogenies^45,46^, and time calibrated our trees using Bayesian fossil-calibrated divergence time estimation in BEAST^47,48^ (Supplementary Fig. 1). Having obtained time-calibrated estimates of branch lengths for all trees in the posterior distribution of our divergence time estimation analysis, we used Markov models to estimate ancestral states of spatial regulatory gene deployment. We estimated ancestral states at every internal node of our phylogeny on a random sample of 10,000 trees from our posterior in a Bayesian framework^42^. Analyses were carried out using the Bayestraits wrapper in R (see “Methods”; http://rgriff23.github.io/projects/btw.html). By reconstructing ancestral states in a Bayesian framework, we explicitly accounted for differences in branch lengths between our 10,000 posterior trees, and integrated over uncertainty in these branch lengths^42^. We performed analyses under a number of different models, as well as with different priors on model parameters, and results were broadly the same regardless of model or prior choice (Supplementary Figs. 2–11; Supplementary Tables 1–26). Each analysis was run for 10,000,000 generations sampling every 1000th generation, allowing us to determine the most probable spatial gene expression pattern present at ancestral nodes. Mean posterior probabilities (PPs) plotted at each node of our phylogeny indicate the probability of a gene expression pattern character state. Throughout the manuscript and in Figs. 2 and 3, we refer to mean PPs derived from analyses resulting from a single-rate model with a Uniform prior on transition rates from 0 to 2.

Considering parsimony alone, plotting the presence of larval skeletogenic cells on the most recent phylogeny of eleutherozoans (Fig. 1b) suggests two evolutionary scenarios for the origin of larval skeletogenic cells: either two gains occurred (one in ophiuroids and one in echinozoans); or one gain occurred at the base of eleutherozoans with one loss in the stem lineage leading to asteroids. To inform these competing evolutionary hypotheses, we performed ancestral state reconstruction on spatial gene expression patterns in early development for four regulatory molecules with detailed functional roles during the specification of larval skeletogenic cells: *alx1*^9–11,13,15,18,20,49–54^, *erg*^17,20,55,56^, *ets1*^11,13,15,18,19,50–53,56–59^, *tbrain*^7,8,10,11,13,15,18,20,52,59–65^, and *vegfr*^17,31,66–68^.

### Ancestral state reconstruction of skeletogenic cell identity

At all nodes of our phylogeny, we found support for spatial gene expression of *alx1* specifically in larval skeletogenic cells, as well as support for broad spatial expression of *ets1* in mesoderm (Node 1; PPs = 0.98) (Fig. 2; Supplementary Figs. 3, 4, 7, and 8; Supplementary Tables 2, 3, 7, and 8). These results suggest that the ancestral state of eleutherozoan echinoderms is likely more similar to states seen in extant echinozoans and ophiuroids rather than those observed in asteroids. Similar to results for *ets1*, though with fewer taxa, ancestral state reconstruction of *erg* showed support for broad expression in mesodermal cell types in early development since the divergence of eleutherozoans (Nodes 1–4; PPs ≥ 0.96) (Fig. 3a; Supplementary Figs. 5 and 9; Supplementary Table 5). Ancestral state reconstruction for the signaling receptor *vegfr*, which is critical both for specification and spatial positioning of skeletogenic cells, showed support for skeletogenic specific spatial expression at the most recent common ancestor (MRCA) of eleutherozoans (Node 1, PP = 0.90; Fig. 3b; Supplementary Figs. 2 and 11; Supplementary Tables 6 and 11). However, neither presence nor absence of *vegfr* in skeletogenic mesoderm in the MRCA of asterozoans is particularly well supported (Node 5; PP for presence = 0.57) (Fig. 3b; Supplementary Fig. 2; Supplementary Table 6). Similarly, support for *erg* expression in skeletogenic cells or nonskeletogenic mesoderm at this node were equivocal (Node 5; PP = 0.5) (Fig. 3a; Supplementary Fig. 5; Supplementary Table 5) or slightly in favor of echinozoan state if a two rate model is used (Supplementary Fig. 5; Supplementary Table 10). Furthermore, at the ancestral node of asterozoans (Node 10), we find support for *alx1* and *ets1* states observed in extant ophiuroids rather than in asteroids (PPs = 0.57 and 0.56, respectively). As it could be argued this result may be an artifact of small sampling in asteroids relative to ophiuroids and echinozoans, we conducted a hypothesis test using Bayes Factors and found it offered further support for this result (Supplementary Table 27). We also conducted sensitivity analyses on pruned trees to determine if taxonomic sampling biases were skewing results (Supplementary Fig. 12; Supplementary Tables 28–30). These additional analyses suggested that, in the case of *ets1* and *alx1*, inference in the asterozoan MRCA may be biased by taxon sampling, as results of our pruned analyses were equivocal (Supplementary Fig. 12). In contrast, our inferences at the eleutherozoan MRCA are robust to differential taxon sampling (Supplementary Fig. 12). Nevertheless, our phylogenetically expansive analysis suggest that mesodermal expression of *alx1* in early development of asteroids^20^ is likely an asteroid apomorphy, with one possible explanation being that *alx1* participates in other GRN circuits, e.g., basal membrane remodeling and mesenchymal ingression^69^. Conversely, our sensitivity analysis cannot rule out a reversal back to the ancestral eleutherozoan state in ophiuroids, though we find it unlikely based on the principle of parsimony. We conclude that *alx1, erg, ets1*, and *vegfr* have been components of larval skeletogenic cell-type identity since its origin in or before the MRCA of eleutherozoans. Furthermore, given the expression of *alx1, ets1*, and *vegfr* in adult skeletogenic cells of asteroids^33^, ophiuroids^70^, and echinoids^33,71^, we conclude that they are likely components of a cell-type identity network possesed by all eleutherozoan skeletogenic cells that likely also drove skeletogenic cell-type identity in ancestral larval skeletogenic cells.

In stark contrast to *alx1*, *erg, ets1*, and *vegfr*, ancestral state reconstruction of the transcription factor *tbrain* revealed marked lability in its spatial deployment at different ancestral nodes (Fig. 2; Supplementary Figs. 6 and 10; Supplementary Tables 4 and 9). We find support for the presence of *tbrain* specifically in ancestral larval skeletogenic cells of camarodont euechinoids included in our analysis (Node 5, PP = 0.96), suggesting it functions specifically in the specification of larval skeletogenic cells and is an apomorphy of euechinoids. Moving deeper in evolutionary time to the divergence of the echinoids about 300 million years ago, there is support for *tbrain* functioning throughout the mesoderm (Node 3, PP = 0.82). At the echinozoan MRCA (Node 2), we also have support for mesodermal *tbrain*. This results stands in contrast to broader endomesodermal expression patterns observed in asteroids^61,63^ and hemichordates^72^, though we note that with inclusion of hemichordate outgroups in the future this result could change. Our results suggest that in the MRCA of echinoids, *tbrain* functioned more broadly in specifying mesodermal cell-type identities as it does in cidaroid echinoids and holothurians today, and that in the lineage leading to camarodont euechinoids, *tbrain* lost its functional role in non-skeletogenic mesodermal cell-type identity but maintained its derived function within the skeletogenic cell-type identity network. Thus, in contrast to an ancestral *tbrain* endomesodermal expression pattern, our analyses support an ancestral mesodermal expression pattern of *tbrain* with gains of expression in endodermal cells, although it must be noted that the PP for this node is relatively low.

## Discussion

To inform the evolution of the echinoderm larval skeletogenic cell, we have presented a framework for cell-type phylogenetic analysis that integrates spatial gene expression data with phylogenetic comparative methods to reconstruct ancestral gene expression. The genes we chose to include in our analyses have been studied in numerous echinoderm taxa and occupy crucial nodes of a well characterized gene regulatory network^11,35^. As the number of model and nonmodel organisms increases in evolutionary developmental biology, comparative analyses of spatial data will depend more on ancestral state reconstructions than on direct comparisons with an outgroup. However, it should be noted that such analyses are limited by several factors, including knowledge of a detailed GRN, invoking interspecies comparisons of development, and obtaining reliable divergence times and phylogenetic trees. The present study is not exempt from these limitations. Indeed, we chose to analyze five genes with broad phylogenetic sampling over twelve taxa. To support our findings, we ran pruned sensitivity analyses and concluded that decreasing the number of taxa reduced our ability to resolve ancestral states with confidence at certain nodes, especially the Asterozoan and Eleutherozoan MRCAs (Supplementary Fig. 12). Therefore, broad phylogenetic sampling is vitally important to resolve ancestral gene expression patterns. One could argue that increasing the number of genes in the analysis would help resolve the question of interest. However, we suggest that whether or not this is true will depend on the case at hand. For instance, in this study we analyzed genes from a GRN where the functional importance of many regulatory genes is well-known. In most cases, a well characterized GRN will not be available, and it will be equally important to possess a broad sampling of taxa across a phylogeny. For instance, as gene expression in early development becomes available in more asterozoans, which are under-sampled with respect to echinozoans in our analyses, we will gain greater confidence in our inferences at the asterozoan and eleutherozoan MRCAs. Indeed, we also see potential for the approach utilized herein to analyze ancestral states of GRN regulatory architecture. For example, the isolation and characterization of homologous *cis-*regulatory modules, from ATAC-seq, ChIP-seq, and other omics-level endeavors in phylogenetically distant organisms, could be then incorporated with the method presented here to determine which modules are lineage or cell-type specific.

**Fig. 4:**
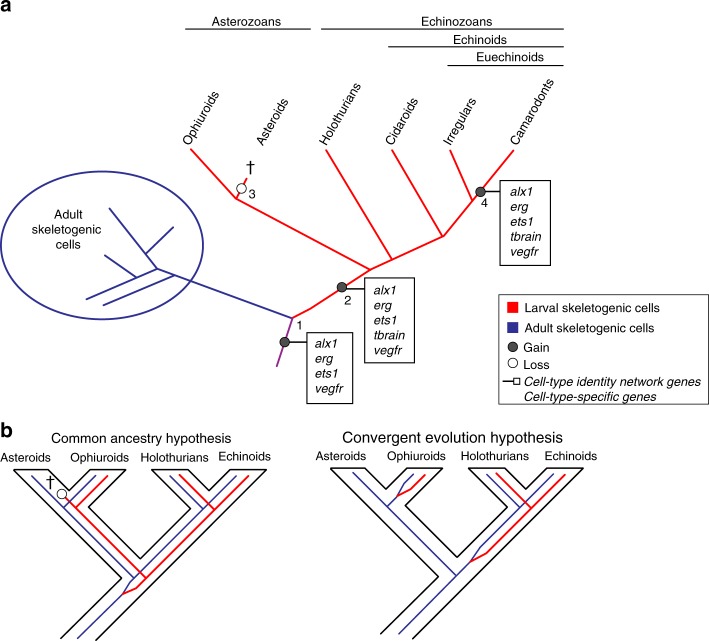
Cell-type evolution of echinoderm larval skeletogenic cells. **a** A cell-type duplication event resulted in activation of the adult skeletogenic cell-type identity network in early development, and thus the presence of skeletogenic cells in ancestral embryonic mesodermal territories (Node 1). *Alx1* and *vegfr* are components of a cell-type specific identity network of larval skeletogenic cells across all extant eleutherozoans. The combination of *alx1, erg, ets1*, and *vegfr* comprise the cell-type identity network of skeletogenic cells across this clade. After the duplication event, at least one transcription factor that was ancestrally expressed in embryonic mesoderm domains, *tbrain*, was subsequently integrated into the larval skeletogenic GRN (Node 2), giving rise to two sister cell types. The larval skeletogenic cell type was lost in asteroids after the divergence with ophiuroids (Node 3). In the lineage leading to extant camarodont euechinoids, *tbrain* was decoupled from and no longer participated in specification of non-skeletogenic mesodermal cell types through Erg-mediated repression. Different colors represent cell-type lineages. Adult skeletogenic cells, purple. Larval skeletogenic cells, red. **b** Two competing hypotheses for the origin of larval skeletogenic cells in eleutherozoan echinoderms. The common ancestry hypothesis suggests that all larval skeletogenic cells in eleutherozoans are the result of a cell-type duplication event in the stem lineage of eleutherozoans. These cells would be lost in the lineage leading to extant asteroids. The convergent evolution hypothesis posits that larval skeletogenic cells are the result of at least two evolutionary events, one in the stem lineage of echinozoans and one other in the stem lineage of ophiuroids. Our results support the common ancestry hypothesis

By framing our results in the context of cell-type evolution, we propose an evolutionary scenario whereby two topographically distinct skeletogenic cell types are the result of a cell-type duplication event that occurred in, or before, the stem lineage of eleutherozoans (Fig. 4a). This event requires heterochronic activation of the adult skeletogenic cell type very early in development, as well as heterotopic activation of the developmental program of adult skeletogenic cells in a distinct developmental context, a similar yet distinct conclusion to a previous analysis^33^. Importantly, our analyses establish continuity of cell-type homology for the echinoderm larval skeletogenic cell, suggesting the existence of a highly conserved cell-type identity network consisting of, but not limited to, the transcription factors *alx1, ets1, erg*, and the signaling molecule *vegfr* that appeared in the stem lineage of eleutherozoan echinoderms, and later was modified when an endomesodermal transcription factor, *tbrain*, acquired functional importance in the larval skeletogenic GRN.

Several observations provide a roadmap as to how the cell-type identity networks driving larval skeletogenic cells evolved. Our results suggest that *alx1* and *vegfr* are part of a cell-type specific suite of regulatory genes that have been specifically expressed in echinoderm larval skeletogenic cells since the MRCA of extant eleutherozoans (Fig. 4a). Their co-expression in adult skeletogenic cells of asteroids^31,33^, ophiuroids^31,70^, and echinoids^33,71^, as well as the larval skeletogenic cells of ophiuroids, holothuroids, and echinoids, suggest they should be considered cell type-specific regulatory genes for these cell types within eleutherozoans. It has been shown that these two genes are activated by Ets1 in the skeletogenic GRN^11^, which also itself inputs into several skeletogenic differentiation genes (Fig. 1a). However, Ets1 does not activate skeletogenic genes in embryonic domains where it is also active. Another critical regulatory genes in this process is *erg*, which has been shown to be one of the first genes to be activated downstream of *alx1* and *ets1*^11^. Activation of *erg* establishes a subcircuit including *erg, hex*, and *tgif*, which serve to lockdown the regulatory state critical for skeletogenic cell specification. Thus, the combination of Alx1, Erg, Ets1, and Vegfr is a defining feature of the cell-type identity network in both adult and larval skeletogenic cells.

A key evolutionary event underlying the genetic and developmental individuation of adult and larval skeletogenic cells was the integration of *tbrain* into the cell-type identity network of larval skeletogenic cells in the stem lineage of eleutherozoans (Fig. 4a). Tbrain is not expressed in adult skeletogenic cells of asteroids^33^, ophiuroids^70^, and echinoids^33^. As our reconstructions suggest that *tbrain* was likely expressed in ancestral mesodermal cells of the eleutherozoan stem lineage (Fig. 2), we propose that *tbrain* was already present in ancestral eleutherozoan embryonic mesoderm and subsequently was integrated into the larval skeletogenic GRN. One putative mechanism that can accommodate this hypothesis is cell-type fusion, whereby regulatory genes already expressed in ancestral cell types or territories come to be co-expressed in a hybrid cell type^1^. In the case at hand, it would suggest that regulatory modules that respond to and were ancestrally under the control of *tbrain* could have become expressed in the larval skeletogenic cell type. This hypothesis could explain the shallow nature of *tbrain* wiring in the larval skeletogenic GRN (Fig. 1a). It remains to be seen whether other regulatory genes that were ancestrally expressed in embryonic mesoderm may have been integrated in a similar way into the larval skeletogenic GRN. Interestingly our analyses also suggest that *tbrain* subsequently became a cell-type specific component of the larval skeletogenic cells of camarodont euechinoids. Previous observations show that Ets1 protein directly activates the expression of *tbrain* in mesodermal cell types^7,60^. Our analyses provide support for this, as co-expression is considered the ancestral state (Node 3, Fig. 2). Other studies suggest that the restriction of Tbrain to larval skeletogenic cells in camarodont echinoids occurred via upregulation of the *tbrain-*repressor Erg by Ets1 in nonskeletogenic cells. This linkage must be an apomorphy of camarodont euechinoids as co-expression of *erg* and *tbrain* has been shown in cidaroid echinoids^16^, and Tbrain drives expression of *erg* in asteroids^56^. Furthermore, it is clear that *tbrain* has acquired larval skeletogenic cell-type specific functions, as the Tbrain protein sequence has itself evolved distinct DNA binding preferences since the divergence of asteroids and echinoids^73^ and exhibits little overlap of regulation of orthologous genes in early development of these clades^74^. Lastly, it should be noted that, when interpreted within the context of the convergent evolution hypothesis, *tbrain* would have had to be integrated independently into two larval skeletogenic GRNs, i.e., once in ophiuroids and once in echinozoans. Our hypothesis requires only a single *tbrain* integration event in the stem lineage of eleutherozoans.

The evolutionary relatedness of ophiuroid and echinoid larval skeletons, as well as the morphology of the pluteus larva itself, has long been debated in the literature, with various interpretations being given either in favor of convergent evolution^25,26,30,31,35,75^ or in favor of common ancestry^22–24^ (Fig. 4b). These arguments have been based, for instance, on the phylogenetic positions of ophiuroids and echinoids^45^, the developmental and structural differences in skeletal morphology^27^, and developmental gene-expression data^13,29,30,35^. On the other hand, and prior to contemporary resolution of the echinoderm phylogeny, some authors favored a common ancestry hypothesis, which at the time was supported by morphological analyses^24^, paleontological analyses^76^, and phylogenetic analyses^23^. Our results support the common ancestry hypothesis (Fig. 4b). Under this scenario it follows that the lineage leading to extant asteroids lost a larval skeletogenic cell (Fig. 4b). Interestingly, many regulatory genes shown to be important for specification of the euechinoid larval skeletogenic cell are also expressed in early asteroid development, including *alx1*^20^, *ets1*^19,77^, *tbrain*^63,78^, *erg*^56^, *hex*^56^, and *tgif*^56^. However, *vegfr* is not expressed^31,71^. As our analyses show, this suggests that loss of *vegfr*, which is a cell-type specific component of larval skeletogenic cells, during early development in the MRCA of extant asteroids, could have facilitated loss of a larval skeletogenic cell. Lastly, coincident with the evolution of eleutherozoan larval skeletogenic cells was the evolution of distinct isoforms of *alx1* that are specifically expressed in larval skeletogenic cells^49^. These isoforms produce Alx1 proteins that are required for early skeletogenesis. Notably, these isoforms are not expressed early in the development of asteroids although they are present in the genome. This observation suggests a molecular mechanism by which a lineage of echinoderms could lose a larval skeleton, namely by the gain or loss of alternative splicing isoforms early in development^49^. Taken together these observations suggest molecular mechanisms that can rapidly bring about the loss of the larval skeletogenic cells, suggesting that a wholesale loss of larval skeleton in asteroids is a plausible evolutionary scenario.

In regards to the evolution of characters, it can be instructive to ask whether phylogenetic evidence suggests lineages are more likely to gain or lose a character during the course of evolution^79^. Analyses of extant eleutherozoans suggest that larval skeletons have been lost in early development of at least 20 species of echinoids and ophiuroids^80,81^. Surprisingly, vestigial larval skeletons occur frequently in the evolution of echinoderm larval forms^25,82–84^. In indirect developing holothurians, spicules are synthesized in the larva but are not elaborated, suggesting this character was lost or an elaborate skeleton failed to evolve in this lineage^20^. Morphologically speaking, all larval skeletogenic cells observed thus far synthesize triradiate spicules, in spite of the fact that echinoderm skeletogenic cells can readily produce a wide array of spicule morphologies^85,86^. This suggests that a shared developmental pathway may be utilized to synthesize early spicules in all lineages of echinoderms that possess larval skeletogenic cells. While our analyses do not strongly refute the convergent evolution of larval skeletogenic cells or the structures they synthesize, we only wish here to point out that future studies give due consideration to both evolutionary scenarios while undertaking comparative work analyzing the evolution of these structures and cell types, an argument that has also been suggested by others^31,87^.

It has been stated that heterochrony tinkers, but heterotopy creates^88^. Whereas heterochrony shifts developmental events in time relative to each other, heterotopy results in the spatial translocation of a developmental structure^89^. The origin of the larval skeletogenic cell is often cited as a classical example of heterochrony. It is also true then that adult skeletogenic cells became housed in a distinct developmental context and were subsequently elaborated into the iconic larval skeleton of echinoids and ophiuroids. In our view, heterotopy of the larval skeletogenic cell is often overlooked even though it has the potential to yield insight into how this cell-type evolved. Our cell-type duplication model predicts that transcription factors and signaling systems that were expressed in ancestral endomesodermal domains were likely integrated into the nascent larval skeletogenic cell in stem eleutherozoans. Such a model may help guide future interpretations of how these cell types evolved. Whether the elaborate larval skeletons of echinoids and ophiuroids are convergent or a product of common descent, it is clear that the distinct differences in morphology, development, and even families of differentiation genes underlying these two morphological structures had hundreds of millions of years to evolve. Therefore, the differences we see today may in fact be a product of the subsequent evolution that has occurred since the divergence long ago of these clades. To understand the evolutionary relatedness of these cells and of these morphological structures, we will need to bring to bear all tools at our disposal. By reconstructing character states driving changes in cell-type identity networks and ancestral cell types, cell-type phylogenetics is one such tool and has the capacity to inform very old questions.

## Methods

### Taxon selection, gene selection, and developmental timing

We selected taxa that had published gene-expression patterns during embryogenesis and larval development for five genes, *alx1, erg, ets1, tbrain*, and *vegfr (vegfr-ig-10)*. In addition, these taxa have published 18S and 28S small subunit ribosomal RNA gene-sequence data which allowed us to time-calibrate our phylogeny using divergence time estimation. Sequences for both loci were available on Genbank for all genera in our analysis except for *Apostichopus*, for which only the 18s sequence was available. Analyses were run for each genus in order to allow us to maximize sampling with respect to these criteria. For instance there were no 28s sequences available on Genbank for *Holothuria leucospilota*, however, sequences for this gene were available for its congener *Holothuria sanctori*. Likewise, despite the fact gene expression patterns have been published for the congeners *Patiria miniata* and *Patiria pectinifera* we include only one asteroid, *Patiria*, in our analyses. In addition, though there is no 18S or 28S gene-sequence data available for the scutellid clypeasteroid *Scaphechinus mirabilis*, sequence data for these two genes were available for *Echinodiscus*, which, as a scutellid, belongs to the same family as *Scaphechinus*. For divergence time estimation, we thus used the sequence data from *Echinodiscus* to time calibrate the divergence between the neognathostomate and atelostomate irregular echinoids, which represents the divergence between *Scaphechinus* and *Echinocardium* in our analyses. For *alx1* and *ets1* sampling was good, and we were able to include twelve taxa in our analyses (Supplementary Data 1). For *tbrain*, we included all of the same taxa as for *alx1* and *ets1* except for the ophiuroid *Amphipholis*, for which there are no published expression patterns for *tbrain*. For *vegfr*, sampling is more limited, and gene expression data were available for seven genera, while for *erg* data were only available for six.

For gene selection, the number of genes included in the GRN for echinoderm skeletogenesis is large, and includes numerous transcription factors and even more differentiation genes. However, this GRN only pertains to euechinoid sea urchins, as this clade is the exclusive source of data for the GRN. We aimed to include genes where the spatial distribution of RNAs is known in both the adult and the early embryo in as many clades of echinoderms as possible. There are very few genes in the GRN where data both in the adult and larva exist in numerous taxa. We included *alx1, erg, ets1, tbrain*, and *vegfr* in our analyses mainly due to the spread of available data. For the other genes, data are piecemeal throughout developmental time and the phylogenetic tree. Thus we aimed to provide an analysis that included as many genes as possible for which there exists a phylogenetically broad sampling. Secondly, as far as specification of skeletogenic cells is concerned, it is clear from developmental studies that these four genes lie at key nodes of the GRN. On the other hand, we included *tbrain* due to its long known activity in different embryonic domains. This gene was included to provide an intriguing contrast to the other selected genes, which, relatively speaking, show domain-specific expression across all taxa analyzed.

Developmental timing varies across taxa, and careful selection of developmental timepoints is critical to obtain meaningful inter-taxon comparisons. In this study, we combined descriptions in the primary literature with analyses of spatial expression patterns to determine where a given gene was expressed. With the exception of sea stars, *alx1* and *vegfr* are in all taxa expressed specifically in skeletogenic cells; and we used their activation and stabilization in skeletogenic precursors as a comparable developmental stage (e.g., mesenchyme blastula in euechinoids) for analysis in each taxon. We used this timepoint as a basis of developmental comparison, and thus collated spatial expression patterns for *ets1, tbrain*, and *erg* at these timepoints in each taxon. Broadly, these criteria resulted in the following developmental timepoints: mesenchyme blastula for euechinoids and ophiuroids, and late blastula/early gastrula for cidaroids, asteroids, and holothuroids.

### Divergence time estimation

So that branch lengths of the phylogenetic trees onto which we reconstructed ancestral states reflected evolutionary time, we used fossil calibrated divergence time estimation to calibrate our phylogenetic tree. For our divergence time estimation analyses, we used two genes, 18S and 28S small subunit ribosomal RNA genes, which comprised the most complete dataset with respect to number of sites for the taxa included in our analyses. Sequences were concatenated and aligned using Clustal X^90^. The aligned matrix is 2919 base pairs long and is available in Supplementary File 1 at https://github.com/jthechino/Erkenbrack_-_Thompson. Each gene was treated as a separate partition, and for each of our two partitions, the best fitting model was determined using the Akaike Information Criterion in JmodelTest 2.0. For both partitions, the best model was identified as the GTR + I + Γ and the GTR + Γ identified as the second best model. Due to statistical issues associated with invariant sites^91^ we performed our analyses using the GTR + Γ model with four gamma categories.

Our divergence time analyses were run using the BEAST 2.3^47^ software package using a constraint tree topology based off of recently published echinoderm phylogenies^45,92^ and calibrated using the seven constraints from the echinoderm fossil record (see “Calibration justification in divergence time analyses”). We used a relaxed clock lognormal model where the substitution rate at each branch is an independent draw from a lognormal distribution with parameters *µ* and *σ*^247^. To set priors on the parameters of our clock model, we first estimated the substitution rate per time unit with a strict molecular clock using the program BASEML in PAML^93^ and the root node calibrated at 500 MA. The substitution rate was found to be 0.016 + −0.001. This was used to calculate the parameters *α* = 1 and *β* = 62.5 of the gamma distribution used as the prior on the parameter *µ* following *α* = (0.016/0.016)^2^ and *β* = 0.016/(0.016^2^). The prior on *σ*^2^ was a gamma distribution with parameters *α* = 1 and *β* = 5. We used a birth-death prior on tree shape with a uniform prior of U(0,10000) on the birth rate. Model parameters were estimated using Markov Chain Monte Carlo (MCMC) for 20,000,000 iterations, sampling every 100th generation. Two separate analyses were run, and results were checked for convergence using the Tracer version 1.6 software^94^. The time tree showing 95% credible intervals on divergence times is shown in Supplementary Fig. 1 with 20% of the posterior sample discarded as burn in. A.xml file with details of analyses is in Supplementary File 1.

### Ancestral state reconstruction

Ancestral state reconstruction models character evolution, in this case the evolution of gene expression patterns, as a Markov process, where the probability of an evolutionary change from one state to another is independent along each branch and depends only on the state at the beginning of each branch^95–97^. In reconstructing ancestral states, this method not only takes into account the topological relationships of taxa, but also branch lengths on the phylogeny. Because of uncertainty regarding the divergence times of the taxa included in our analyses, and thus uncertainty in branch lengths in our phylogenies, we used a Bayesian approach to explicitly integrate over this uncertainty^42^. We thus used a random sample of 10,000 time-calibrated trees from the posterior distribution of our divergence time estimation analyses as the input trees for our ancestral state reconstructions. For ancestral state reconstructions, we used the program BayesTraits V.3^42^ run in the R software environment using the wrapper program btw (http://rgriff23.github.io/projects/btw.html)^98^.

For all analyses, branch lengths were scaled to have mean value of 0.1 as recommended by the BayesTraits manual. All analyses were initially run with a uniform prior between 0 and 1 on transition rates. Inspection of posterior distribution showed that this prior was truncating the posterior distribution, so analyses were re-run with a uniform prior between 0 and 2. For *alx1, ets1, erg*, and *vegfr*, characters were all scored as binary traits; *tbrain*, which displays a diversity of expression patterns throughout extant echinoderms, was scored as a multistate character with four character states. For all binary traits, the logarithm of the marginal likelihood was computed for each model using stepping stone sampling^99^ and the 2 times the Log Bayes Factor^100^ was computed to compare support for a single rate model, where q_01_ and q_10_ were constrained to be equal, and a two-rate model where each rate was allowed to take its own value^101^. For *tbrain*, Log Bayes Factors were computed to compare a single rate model to a multi-rate model. Results of Bayes Factor comparisons between one rate and multiple rate models are shown in Supplementary Table 1 and in all cases support one model over the other was negligible. Furthermore, analyses did not differ when analyses were run using either a one rate or multi-rate model (see “Sensitivity analyses”).

Bayesian ancestral state reconstructions use MCMC to estimate model parameters, and in particular the instantaneous transition rate for each character being analyzed. MCMC was run for 10,000,000 generations sampling every 1000 generations. Burn-in was 2,000,000 generations. Convergence was assessed by plotting and inspecting the sampled value per iteration, the probability density function for the posterior, and the autocorrelation for the Markov chain of the transition rate q_01_ using the traceplot(), densplot(), and autocorr.plot() functions in the CODA package^102^ in R. Results of ancestral state reconstructions using a single rate model are shown in Supplementary Tables 2–6 and Supplementary Figs. 2a, 3a, 4a, 5a, and 6a; while analyses using a two-rate, or multi-rate in the case of *tbrain*, are shown in Supplementary Tables 7–11 and Supplementary Figs. 2b, 3b, 4b, 5b and 6b. In most cases, inferred ancestral states did not differ dependent upon whether the model had multiple rates or a single rate. The exception to this was *erg*, where using a two-rate model resulted in slight support for mesodermal expression in the asterozoan MRCA, while the single-rate model returned equivocal results at this node (Supplementary Fig. 5). R code used to perform all analyses can be found at https://github.com/jthechino/Erkenbrack_-_Thompson.

### Sensitivity analyses

In order to explore the sensitivity of our results to changes in model parameters, we ran a number of sensitivity analyses using different priors on the instantaneous transition rate q_01_. We initially used a uniform prior ranging from 0 to 2 on this transition rate (see “Ancestral state reconstruction”). To explore the sensitivity of a more diffuse prior, we ran our analyses with uniform priors of U(0, 20) and U(0, 200). To explore any changes in our results when analyses were run with less-diffuse priors, we ran our ancestral state reconstructions with a uniform prior of U(0, 0.2). Results of these sensitivity analyses are shown in Supplementary Tables 12–26 and Supplementary Figs. 7–11. Running analyses with wider priors reduced the confidence in inferred ancestral states (PPs approached unity at ancestral nodes), though this only slightly changed results for analyses of *ets1* and *alx1*. In the case of these genes inferred ancestral states still supported the presence of an *alx1* + and *ets1* + skeletogenic cell and *ets1* expression in the mesoderm at all ancestral nodes (Supplementary Figs. 7 and 8). Results were similar with respect to *vegfr* and *erg*, though the reduction in PP for all nodes was of greater magnitude (Supplementary Figs. 9 and 11). For *tbrain*, inferred PPs for ancestral states approached unity using the prior of U(0,20), though the inferred ancestral states did not differ from those with a prior of U(0,2), and still showed fairly strong support for the same inferred ancestral states (Supplementary Fig. 10b). The same trend was apparent to a greater extent using a prior of U(0,200), though results at the MRCAs of camarodonts, irregular echinoids, and cidaroids all still showed one state clearly favored over all others (Supplementary Fig. 10c).

Using a narrower prior (U(0, 0.2)) (Supplementary Figs. 7a, 8a, 9a, 10a, and 11a) resulted in slower modeled rates of character evolution, and more definitive inferences for ancestral states. This is not surprising, as lowering the modeled rate of evolution in ancestral state reconstruction analyses results in answers approaching a most-parsimonious reconstruction^97^. As a result, with a narrower prior, the most probable ancestral states inferred were the same as using a prior of U(0,2), but with stronger support for the inferred most likely ancestral states.

In addition to assessing sensitivity of our results to model parameters, we also explored the effects of taxon sampling on our results. This was particularly to assess reconstructions at the MRCA of eleutherozoans, and at the asterozoan MRCA. For ancestral state reconstructions of *alx1*, *ets1*, and *tbrain* we ran pruned analyses including only one taxon per class (four tips total). Results of these analyses are shown in Supplementary Tables 28–30 and Supplementary Fig. 12. In the case of *alx1* and *ets1*, running analyses using only four tips did not alter interpretations at the MRCA of eleutherozoans, which still showed strong support for expression of *alx1* in skeletogenic mesenchyme (Supplementary Fig. 12a), and *ets1* in skeletogenic mesenchyme and non-skeletogenic mesoderm (Supplementary Fig. 12b); however, inferences at the MRCA of asterozoans were equivocal for both these genes (Supplementary Fig. 12a, b). Likewise, for *tbrain*, using only for tips resulted in ambiguous resolution at the MRCA of eleutherozoans, and ambiguous inferences at the MRCA of asterozoans and echinozoans (Supplementary Fig. 12c).

### Bayes factor hypothesis testing

In order to compare support for different reconstructions of ancestral expression patterns for *alx1*, *ets1, erg*, and *vegfr* at the MRCA of asterozoans, we fixed the value at this node as either 0 or 1, corresponding to either expression in the mesoderm for *alx1, erg*, and *ets1* or expression in the skeletogenic cell or NSM and skeletogenic cell for *ets1* and *erg* or *alx1*, respectively. Bayes factors were then computed to estimate support for different models estimated using these fixed values. 2*ln Bayes Factors were calculated as 2*(ln[*P*|State = 0] − ln[*P*|State = 1] where ln[*P*|State] is the natural log of the marginal likelihood of the model calculated when the node representing the MRCA of asterozoans is fixed at either 0 or 1. Results were interpreted following the tables reported in Kass and Raftery^100^. Natural log marginal likelihoods were calculated in BayesTraits with the btw wrapper using stepping stone sampling^99^. MCMC for stepping stone sampling was run for 1000 stones with 100,000 iterations per stone. Bayes factors resulting from comparisons for each gene are shown in Supplementary Table 27. For both *alx1* and *vegfr*, hypothesis testing using Bayes factors supported expression in the skeletogenic mesenchyme. For *ets1* and *erg* Bayes factors supported the expression in the skeletogenic mesenchyme and nonskeletogenic mesoderm.

### Calibration justification in divergence time analyses

Euechinoid-Cidaroid divergence—The divergence between cidaroid and euechinoid echinoids represents the oldest occurrence of a crown group echinoid in the fossil record, and thus the hard minimum on the divergence of the echinoid crown group. The oldest crown group echinoid is *Eotiaris guadalupensis* Thompson, 2017 from the Roadian of the Road Canyon Formation of West, Texas, USA^103,104^. Thompson et al.^104^ found this taxon to be a cidaroid using phylogenetic analyses, and the presence of two columns of interambulacral plates in an interambulacral area and a perignathic girdle of apophyses readily classify this species amongst the cidaroidea. The exact stratigraphic distribution of *E. guadalupensis* within the Roadian is unknown, so the top of the Roadian stage, 268.8 MYA, is used as the hard minimum for the divergence of the euechinoids and cidaroids. We round this to 269 for purposes of divergence time estimation. The maximum bound used for calibration is the bottom of the Viséan stage, or 346.7 MYA. The Viséan is home to abundant and diverse echinoid faunas including those of the Edwardsville Formation^105^, the Fort Payne Formation^106^, and the Molignée Formation^107,108^. The diversity and abundance of stem group echinoids in these faunas provide a taphonomic control; and despite the number of stem group echinoids known from these deposits, crown group echinoids are wanting, and thus the bottom of the Viséan is used as the maximum bound on the divergence.

Irregularia-Camarodonta divergence—The oldest irregular echinoid, *Jesionekechinus hawkinsi*, from the Sunrise Formation of New York Canyon, Nevada^109^ calibrates the hard minimum of the divergence between the Irregularia and the camarodont echinoids. *J. hawkinsi* was recorded from 15 to 30 ft below the *Eoderoceras* zone, which is equivalent to the unit G of Hallam^110^, which is the base of the Pleinsbachian Joker Peak member^111^. If *J. hawkinsi* is from below the Joker Peak member, at the youngest it must be from the New York Canyon Member, which underlies the Joker Peak Member. The age of the New York Canyon Member is Sinnemurian^111^, and given the imprecise stratigraphic position of *J. hawkinsi*, we use the top of the Sinemmurian, 190.8 MYA, as the hard minimum on the divergence between camarodonts and irregulars. The maximum bound on the divergence is set by the most diverse fauna of echinoids in the Triassic, from the Carnian St. Cassian Formation of Northern Italy^112,113^. This fauna is currently interpreted to consist entirely of cidaroids; though there are specimens with euechinoid-like morphologies known from disarticulated material, there are no putative irregulars. The age of the Cassian Formation is Carnian, and spans from the Julian 1 slightly into the Julian 2 ammonoid zones^114^. We use the top of the Julian 1 ammonoid zone as the lower constraint on the Irregularia-Camarodonta divergence, which is approximately 235 MYA.

Holothuroid-Echinoid divergence—There are a number of basal fossil holothuroids and echinoids in Ordovician strata^115,116^. The divergence between these two clades is calibrated by the oldest unequivocal fossil holothurian calcareous ring elements and body wall ossicles which are from the Red *Orthoceras* limestone of Sweden, which was found as a glacial erratic boulder in northern Germany^116^. These specimens were recovered from the *Eoplacognathus suecicus* conodont zone, which is itself within the *Pseudoclamacograptus decorates* graptolite zone. The top of the *P. decorates* zone is 463.97 MA, which we round to 464 MA for purposes of divergence time estimation. The maximum bound on the divergence is set by the Fezouata Lagerstätte, which yields a diverse and abundant echinoderm fauna^117^. Despite the wealth of echinoderms from the Fezouata, there have been no echinoid or holothurians fossils recovered. The Fezouata Shale is at the youngest Floian^117^, and thus we use the top of the Floian stage of the Ordovician as the maximum bound on this divergence, which is 470 MA.

Neognathostomata-Atelostomata divergence—*Echinodiscus* is a clypeasteroid echinoid, which is part of the larger clade the Neognathostomata. The clypeasteroids did not evolve and diversify until the Cenozoic^92^; however, they are paraphyletic with respect to the morphologically conservative cassiduloida^92^. Kroh and Smith^92^ found the *Galeropygus*, the Nucleolitidae, and the Clypeidae to be stem group neognathostomates but with low-bootstrap support. Barras^118^ additionally found the nucleolitids and clypeids to be amongst the neognathostomates (what he referred to as cassiduloids) in his 50% majority rule consensus tree. The stem group of the atelostomates comprises a number of species and genera previously known as the disasteroids. The phylogenetic position of these taxa seems to be sensitive to character choice and weighting scheme^92,118^. We thus use the first occurrence of stem neognathostomates in the fossil record to calibrate the minimum divergence between the atelostomates and neognathostomates. The oldest clypeid is *Clypeus rostratus* from the Toarcian *D. levesquei* ammonite zone of the Upper Lias of Walditch, Dorset. Barras^118^. The *D. levesquei* Zone is roughly equivalent to the *D. pseudoradiosa* and *P. dispansum* zones. The top of the *D. pseudoradiosa* zone is 174.71 MA. We round this to 174 MA, which we use as the minimum constraint on the Neognathostomata-Atelostomata divergence. The lower Jurassic has a rich echinoid fauna, however, irregular echinoids are rare. We thus use the base of the Jurassic, 201.3 MA, as the maximum bound on the neognathostomate and atelostomate divergence, which we round to 201 MA.

Asterozoan-Echinozoan divergence—Asterozoans are known from much earlier in the Ordovician fossil record than are crown-group echinozoans, and thus the divergence between asteroids and ophiuroids is calibrated by the oldest asterozoan known from the fossil record, *Maydena roadsidensis*^119^. *M. roadsidensis* co-occurs with the graptolite *Psigraptus jacksoni* in the *Psigraptus* zone, the top of which is dated as 481.67 MA. We round this to 481 MA, which is used as the hard minimum on the divergence of the asterozoans and echinozoans. The phylogenetic relationships amongst the early eleutherozoans are not well constrained, and thus the ancestry of echinozoans and asterozoans is unclear^120^. Echinozoans and asterozoans may have both evolved from an edrioasteroid ancestor, but though which edrioasteroids they evolved from are unknown. In order to account for this uncertainty, we use the oldest edrioasteroid, *Stromatocystites walcotti*, from the Cambrian Series 2^121^ as the maximum bound on the divergence. The maximum bound on the divergence is thus the base of Series 2 in the Cambrian Period, which is currently set at 521 MA.

Asteroid-Ophiuroid divergence—The asterozoans include ophiuroids, asteroids, and the extinct Early Paleozoic group the somasteroids^122^. As discussed above, the earliest asterozoan is *Maydena roadsidensis*^119^, which was noted by Jell^119^ as “likely to be part of the lineage leading to earliest ophiuroids such as *Pradesura* Spenger, 1951 and *Eophiura* Jaekel, 1903” (Jell^119^ p. 536). The ophiuroid *Pradesura jacobi* is the geologically oldest nonsomasteroid asterozoan other than *M. roadsidensis*^119^. *P. jacobi* is known from the St. Chinian Schist Formation, northeast of St. Chinian, la Croix-Rouge, southeast France^122^. While the phylogenetic position of *M. roadsidensis* has yet to be examined rigorously, *P. jacobi* was found to form a clade with other basal ophiuroids by Shackleton^122^. The age of *P. jacobi* is latest Tremadocian^119^. We thus use the top of the Tremadocian, 477.7 MA (rounded to 477 MA), as the hard minimum on the divergence between asteroids and ophiuroids. As previously mentioned, asterozoans can trace their ancestry to edrioasteroids. As for the Asterozoan-Echinozoan divergence, we thus use the occurrence of *Stromatocystites walcotti* and the base of the Cambrian Series 2, 521 MA, as the maximum bound on the prior for the Asteroid-Ophiuroid divergence.

Holothuriida-Neoholothuriida divergence—*Holothuria* belongs to the holothuroid family Holothuriidae, which was recently demonstrated based upon molecular data to form a clade with the Mesothuriidae, the Holothuriida^123^. The Holothuriida is the sister group to the Neoholothuriida, which contains the genus *Apostichopus*. A thorough discussion of the fossil calibrations for the holothuriidae can be found in Miller et al.^123^. The oldest putative holothuriid ossicles are known from the Wuchiapingian period of the Permian and may represent stem group holothuriids^123^. The oldest known holothuriid calcareous rings are of an undescribed species from the *spinosus* zone of the Early Ladinian Upper Muschelkalk of Baden-Württemberg, Germany^124^. The base of the Ladinian is 241.5 MA^125^ and we use the occurrence of these holothuriid calcareous rings as the hard minimum on the divergence between the holothuriids and neoholothuriids (rounded to 241); while the maximum age for the divergence is taken as Wuchiapingian in age, and is thus treated as 260 MA, approximately the age of the base of the Wuchiapingian^126^.

### Statistics and reproducibility

All code and relevant data to reproduce our analyses are either included in this paper or are available at GitHub https://github.com/jthechino/Erkenbrack_-_Thompson.

### Reporting summary

Further information on research design is available in the Nature Research Reporting Summary linked to this article.

## Supplementary information

Description of Additional Supplementary Files

Supplementary Data 1

Supplementary Information

Reporting Summary

## Data Availability

All data in this study are either available in the Supplement or have been previously published.

## Code availability

Code used to conduct these analyses is available on GitHub at https://github.com/jthechino/Erkenbrack_-_Thompson.
