# Supplementary material for: Cell type phylogenetics informs the evolutionary origin of echinoderm larval skeletogenic cell identity
Source: Commun Biol. 2019 May 3;2:160. doi: 10.1038/s42003-019-0417-3 (PMC6499829; doi:10.1038/s42003-019-0417-3)
Supplement: Supplementary file 1 — Description of Additional Supplementary Files [file 42003_2019_417_MOESM1_ESM.docx]

**Description of Additional Supplementary Items**

Supplementary Data 1. Table showing all of the taxa included in ancestral state reconstructions, the spatial distribution and scoring of gene expression for *alx1*, *erg*, *ets1*, *vegfr*, and *tbrain,* and references where expression patterns were published. See accompanying Excel spreadsheet.
