# Supplementary material for: Cell type phylogenetics informs the evolutionary origin of echinoderm larval skeletogenic cell identity
Source: Commun Biol. 2019 May 3;2:160. doi: 10.1038/s42003-019-0417-3 (PMC6499829; doi:10.1038/s42003-019-0417-3)
Supplement: Supplementary file 3 — Supplementary Information [file 42003_2019_417_MOESM3_ESM.pdf]

Supplementary Figures

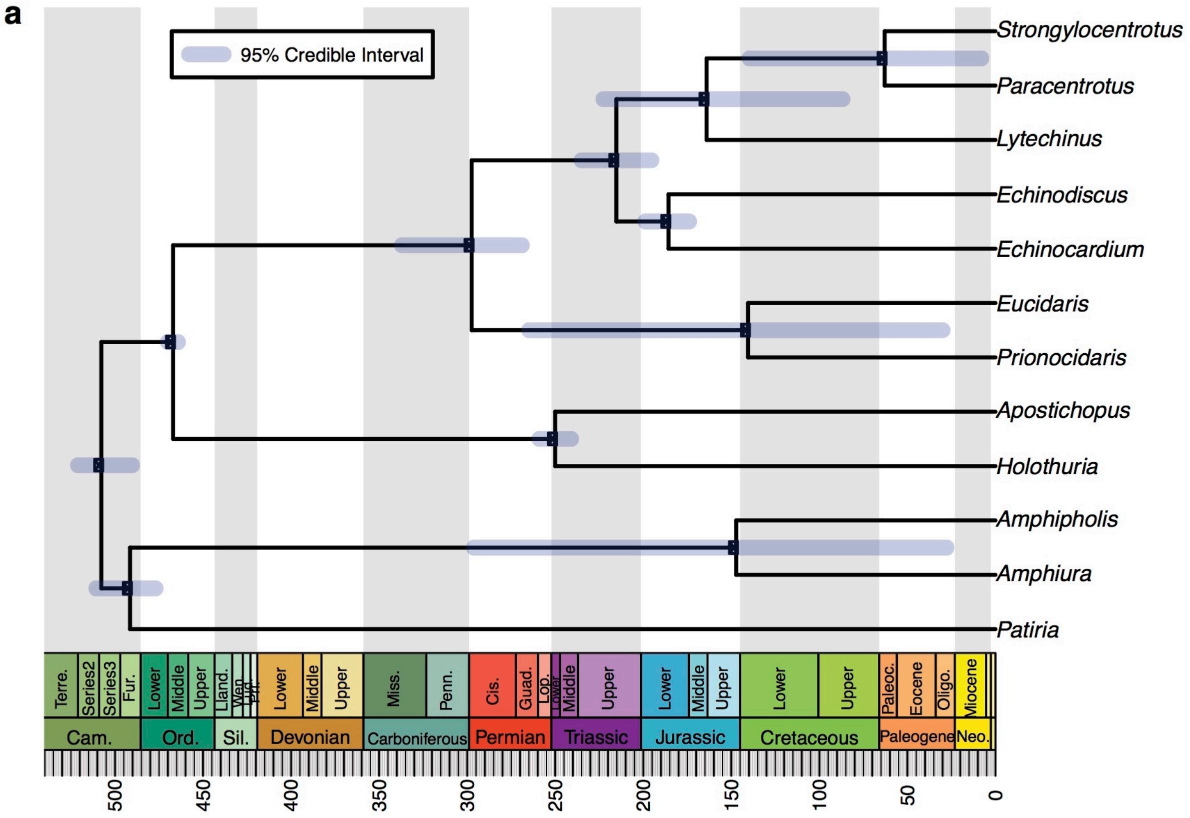

Supplementary Figure 1. Time calibrated echinoderm phylogeny showing 95% credible intervals and mean divergence times for divergences between analyzed taxa. Topology is based on Telford, et al. [1] and Smith et al.[2]. The placement of *Paracentrotus* and *Lytechinus* differ from that of Mongiardino-Koch et al.[3] , but given that these taxa share the same character states in all downstream analyses, the differential placement between that analysis, and the topology used herein, are unlikely to have an impact on our final results. Details of MCMC can be found in Methods under Divergence Time Estimation. Tree is a maximum clade credibility tree constructed from 160,001 topologies sampled from the posterior. Abbreviations are as follows: Cam., Cambrian;Terre., Terreneuvian; Fur., Furongian; Ord., Ordovician; Sil., Silurian; Lland., Llandovery; Wen., Wenlock; Lud., Ludlow; Pri., Pridoli; Miss., Mississippian; Penn., Pennsylvanian; Cis., Cisuralian; Guad., Guadalupian; Lop., Lopingian; Paleoc., Paleoc,. Paleocene; Oligo., Oligocene; Neo., Neogene.

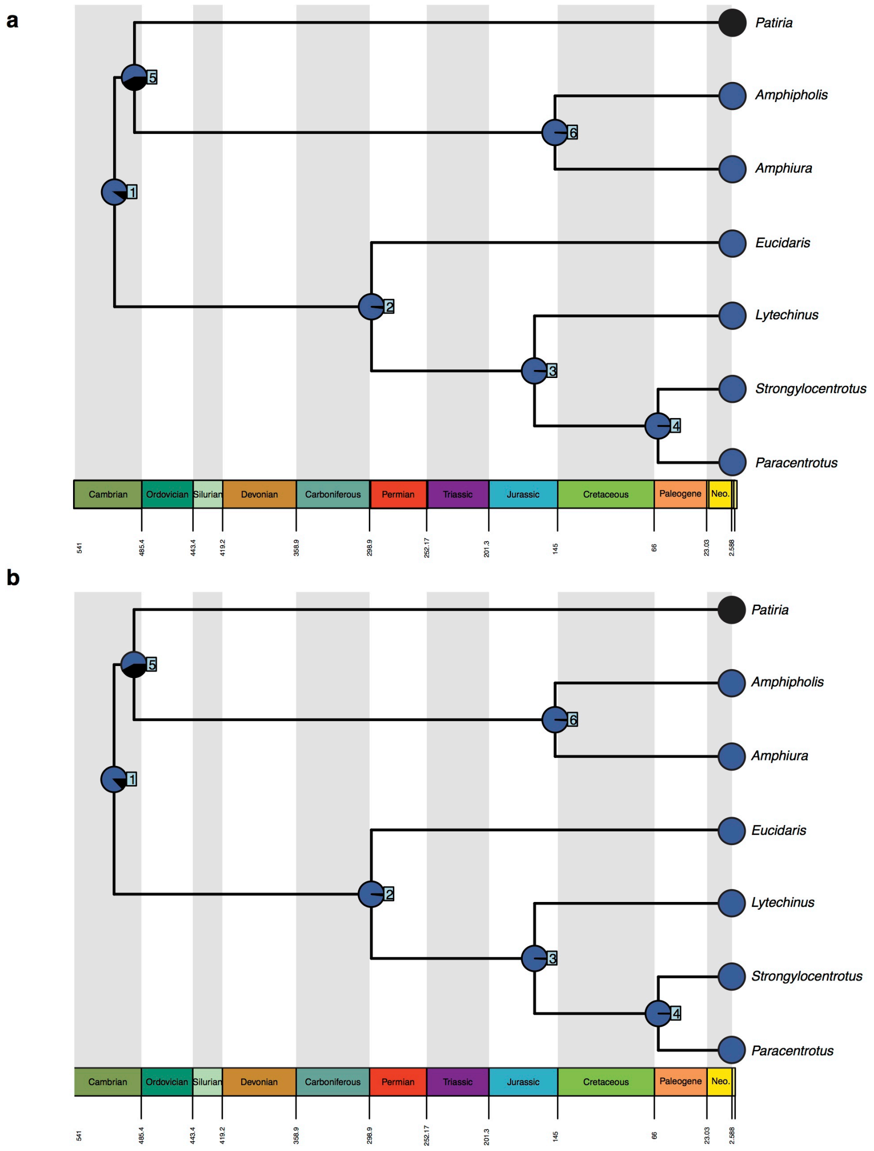

Supplementary Figure 2. Ancestral state reconstruction of *vegfr* gene expression patterns using single-rate and two-rate Markov models. (a) Single-rate Markov model of *vegfr.* Numbers on Nodes correspond to Supplementary Table 6. (b) Two-rate Markov model of *vegfr.* Numbers on Nodes correspond to Supplementary Table 11. Colors are as shown in Figure 1 in the main manuscript. Pie charts represent the mean posterior probabilities from MCMC run using 10,000 trees sampled from the posterior distribution of divergence time estimation analyses.

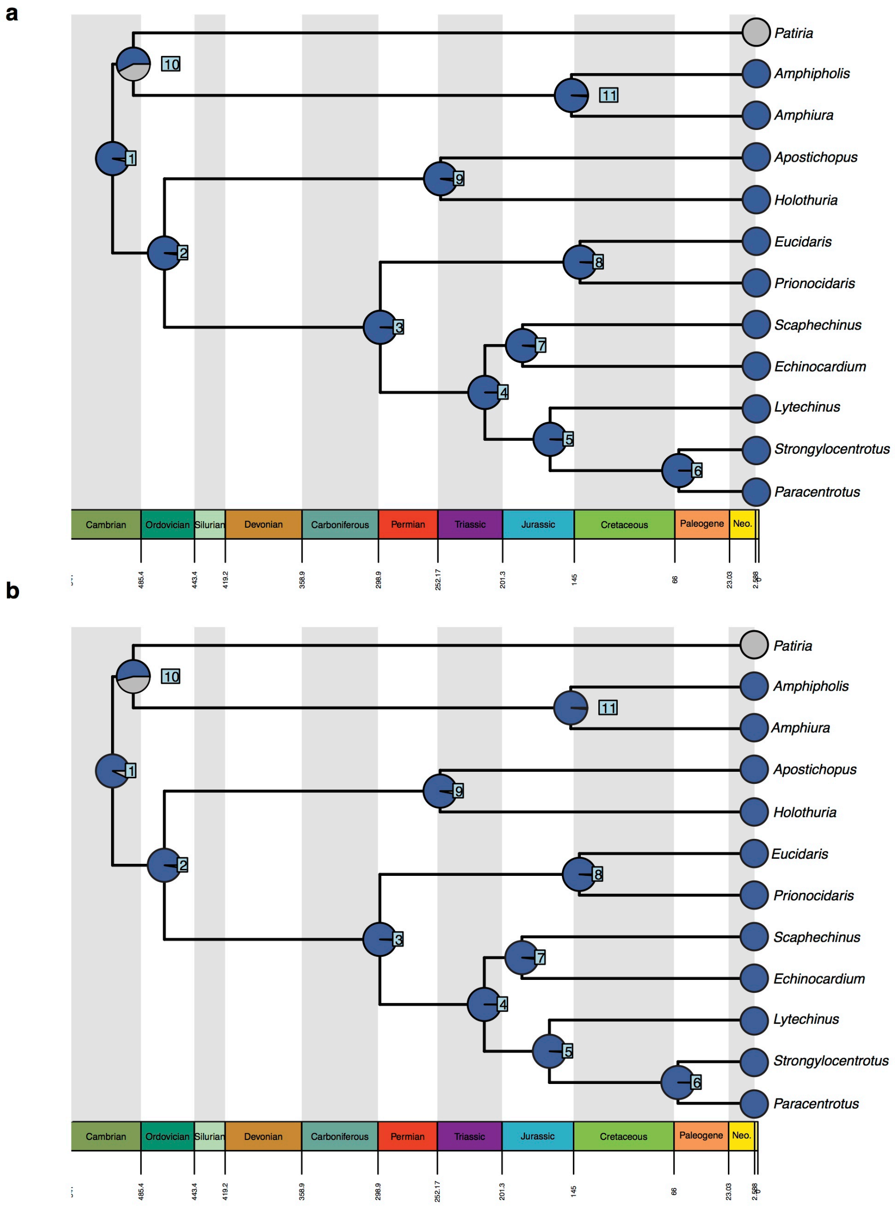

Supplementary Figure 3. Ancestral state reconstruction of *alx1* gene expression patterns using single-rate and two-rate Markov models. (a) Single-rate Markov model of *alx1.* Numbers on Nodes correspond to Supplementary Table 2. (b) Single-rate Markov model of *alx1.* Colors are as shown in Figure 1 in the main manuscript. Pie charts represent the mean posterior probabilities from MCMC run using 10,000 trees sampled from the posterior distribution of divergence time estimation analyses. Numbers on Nodes correspond to Supplementary Table 7.

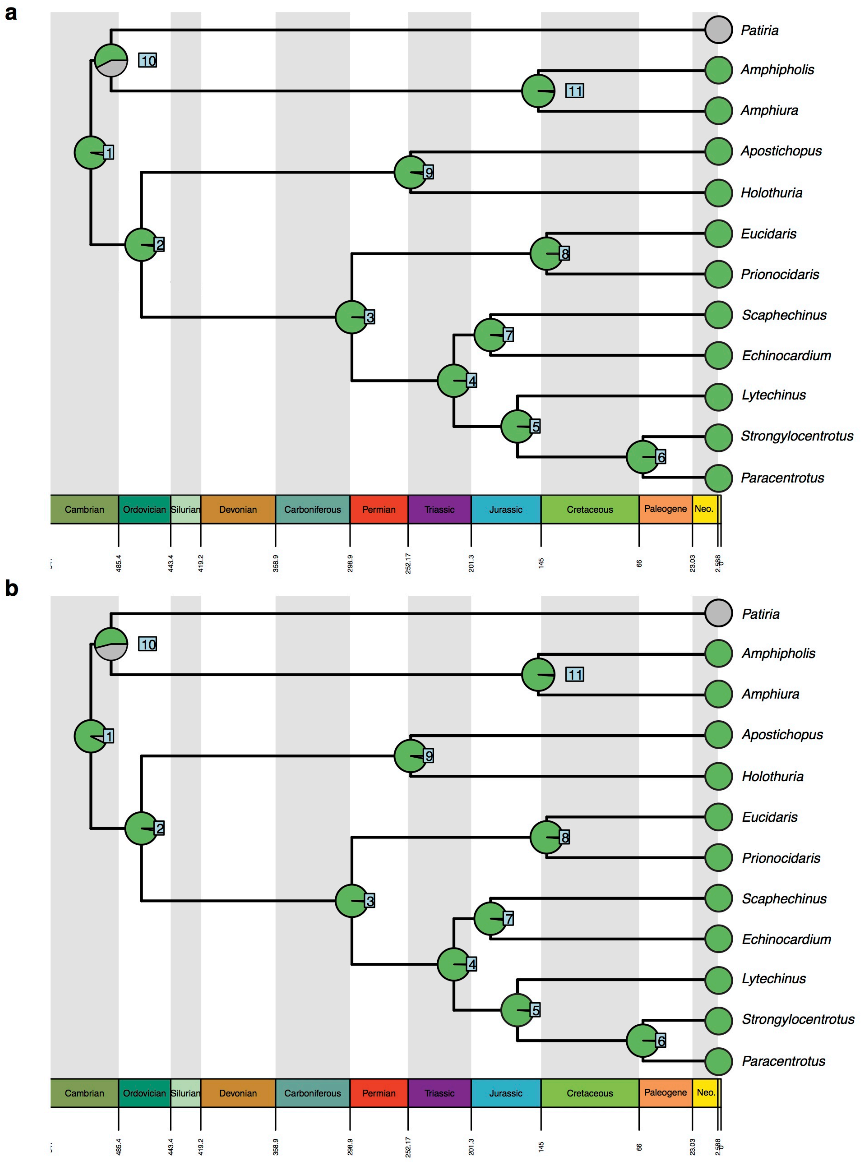

Supplementary Figure 4. Ancestral state reconstruction of *ets1* gene expression patterns using single-rate and two-rate Markov models. (a) Single-rate Markov model of *ets1.* Numbers on Nodes correspond to Supplementary Table 3. (b) Two-rate Markov model of *ets1.* Colors are as shown in Figure 1 in the main manuscript. Pie charts represent the mean posterior probabilities from MCMC run using 10,000 trees sampled from the posterior distribution of divergence time estimation analyses. Numbers on Nodes correspond to Supplementary Table 8.

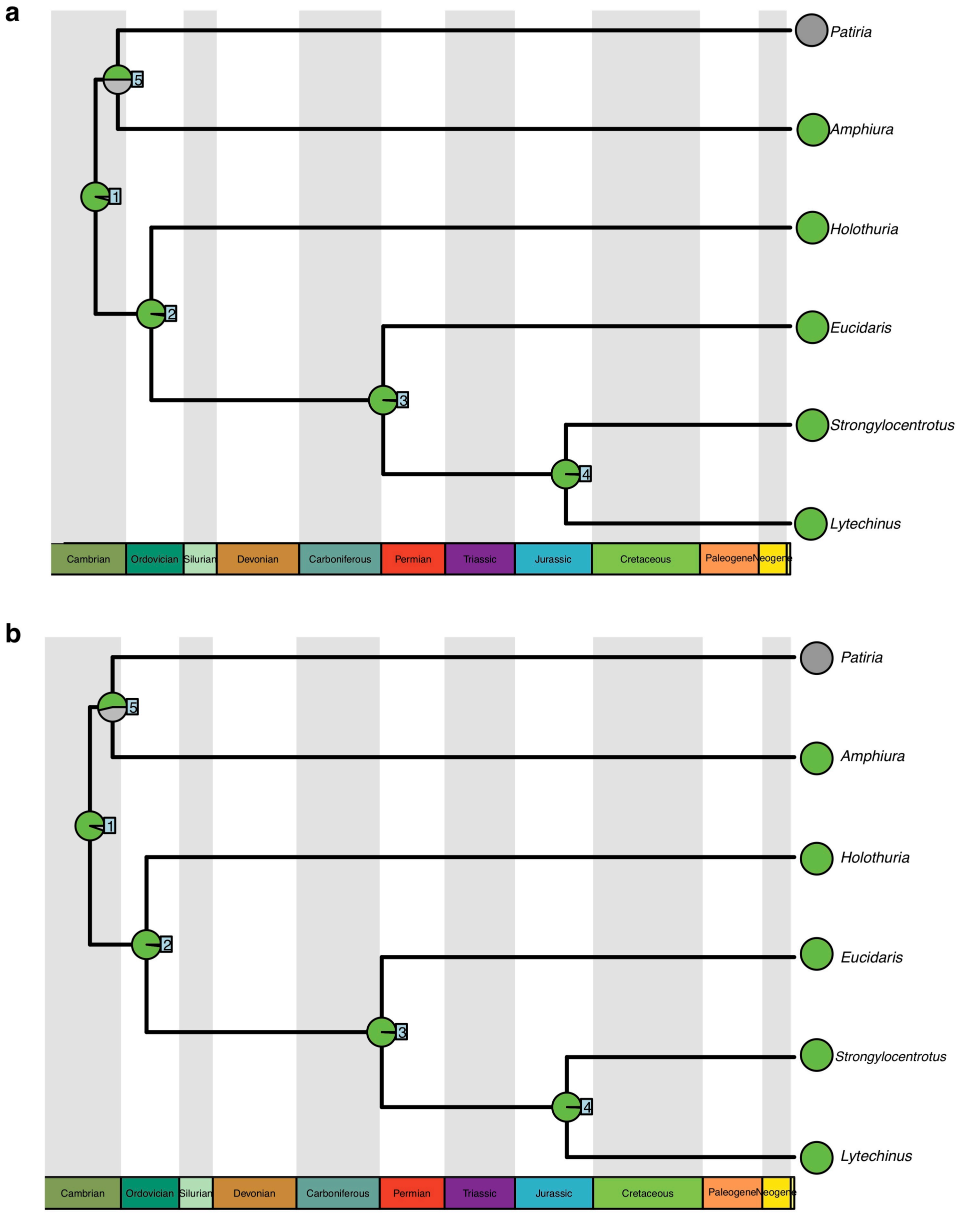

Supplementary Figure 5. Ancestral state reconstruction of *erg* gene expression patterns using single-rate and two-rate Markov models. (a) Single-rate Markov model of *erg.* Numbers on Nodes correspond to Supplementary Table 5. (b) Two-rate Markov model of *erg.* Colors are as shown in Figure 1 in the main manuscript. Pie charts represent the mean posterior probabilities from MCMC run using 10,000 trees sampled from the posterior distribution of divergence time estimation analyses. Numbers on Nodes correspond to Supplementary Table 10.

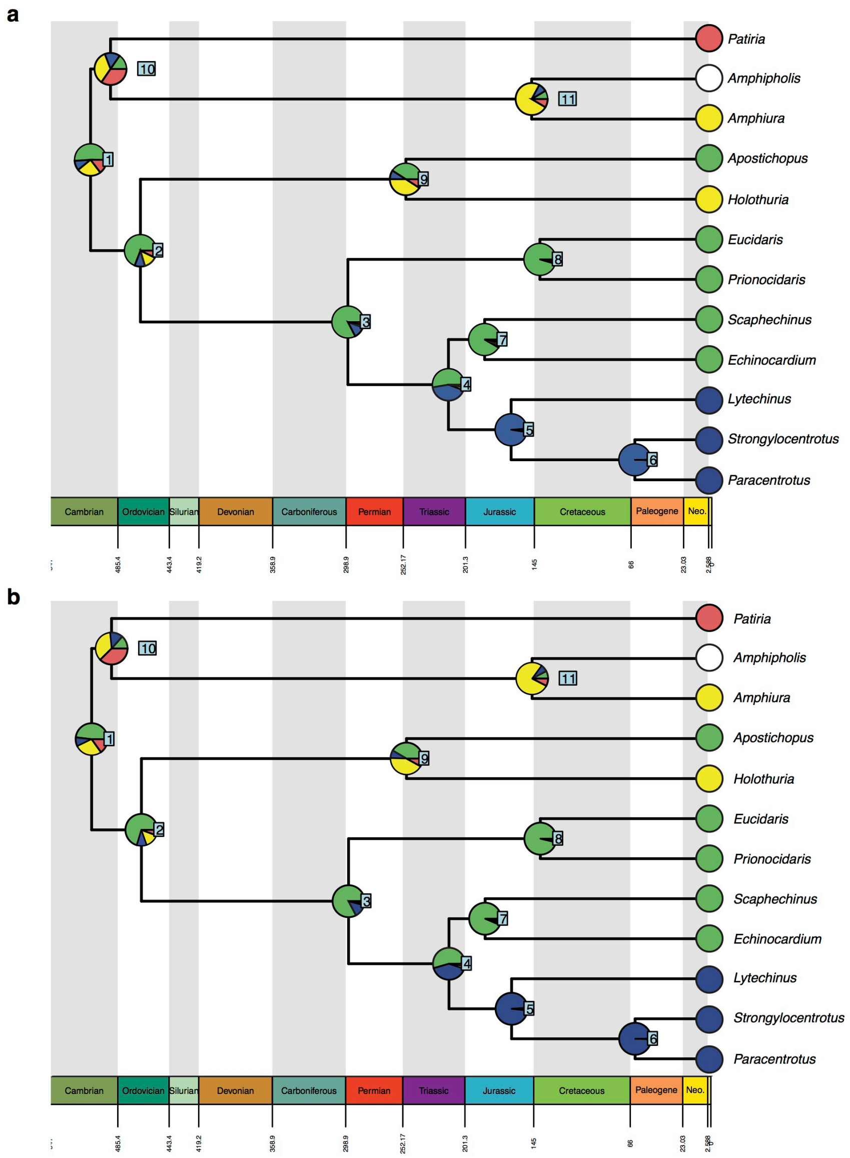

Supplementary Figure 6. Ancestral state reconstruction of *tbrain* gene expression patterns using single-rate and two-rate Markov models. (a) Single-rate Markov model of *tbrain.* Numbers on Nodes correspond to Supplementary Table 4. (b) Two-rate Markov model of *tbrain*. Numbers on Nodes correspond to Supplementary Table 9. Colors are as shown in Figure 1 in the main manuscript. Pie charts represent the mean posterior probabilities from MCMC run using 10,000 trees sampled from the posterior distribution of divergence time estimation analyses.

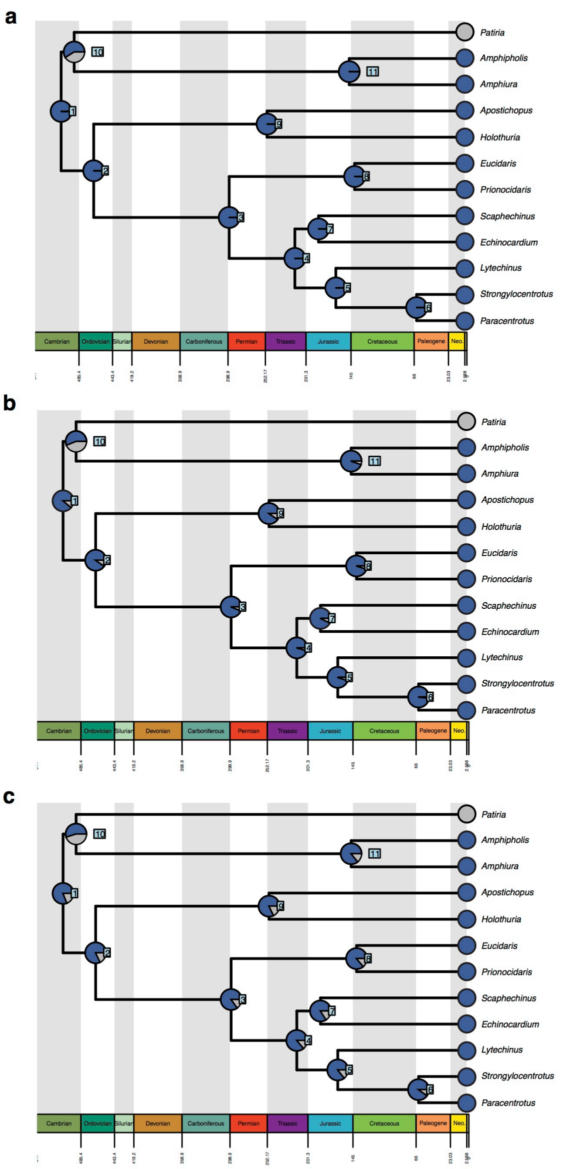

Supplementary Figure 7. Ancestral state reconstruction of *alx1* gene expression patterns using a single-rate Markov model with differing instantaneous transition rates. (a) Prior on the instantaneous transition rate q_{01} set to U(0, 0.2). Numbers on Nodes correspond to Supplementary Table 22. (b) Prior on the instantaneous transition rate q_{01} set to U(0, 20). Numbers on Nodes correspond to Supplementary Table 12. (c) Prior on the instantaneous transition rate q_{01} set to U(0, 200). Numbers on Nodes correspond to Supplementary Table 17. Colors are as shown in Figure 1 in the main manuscript. Pie charts represent the mean posterior probabilities from MCMC run using 10,000 trees sampled from the posterior distribution of divergence time estimation analyses.

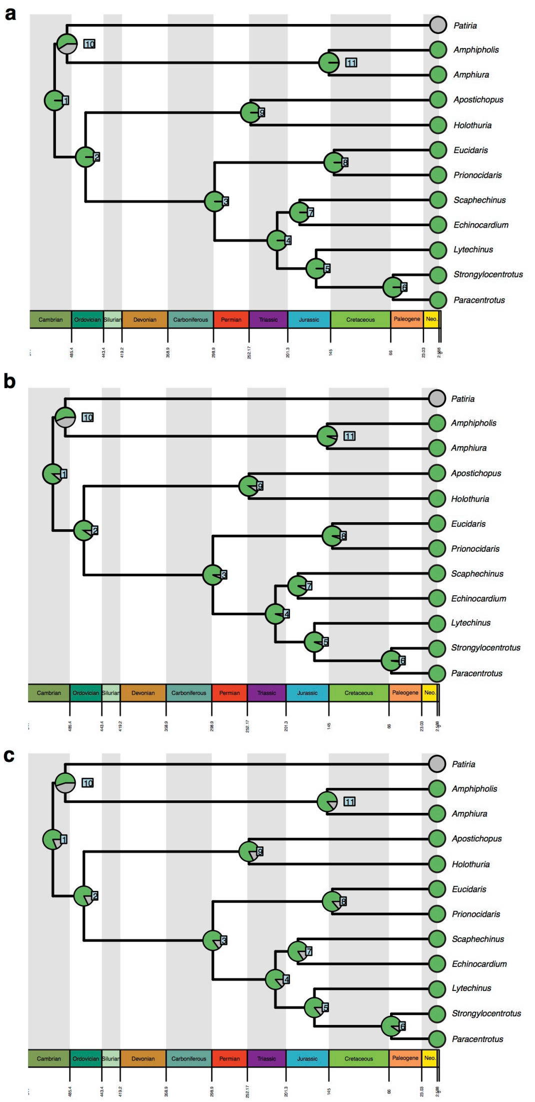

Supplementary Figure 8. Ancestral state reconstruction of *ets1* gene expression patterns using a single-rate Markov model with differing instantaneous transition rates. (a) Prior on the instantaneous transition rate q_{01} set to U(0, 0.2). Numbers on Nodes correspond to Supplementary Table 23. (b) Prior on the instantaneous transition rate q_{01} set to U(0, 20). Numbers on Nodes correspond to Supplementary Table 13. (c) Prior on the instantaneous transition rate q_{01} set to U(0, 200). Numbers on Nodes correspond to Supplementary Table 18. Colors are as shown in Figure 1 in the main manuscript. Pie charts represent the mean posterior probabilities from MCMC run using 10,000 trees sampled from the posterior distribution of divergence time estimation analyses.

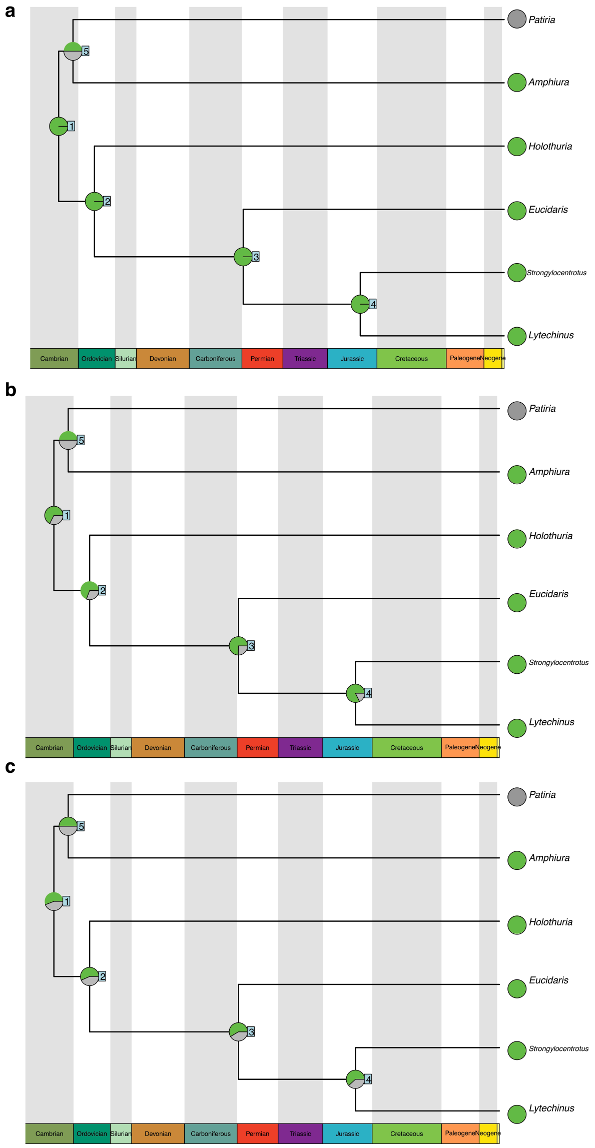

Supplementary Figure 9. Ancestral state reconstruction of *erg* gene expression patterns using a single-rate Markov model with differing instantaneous transition rates. (a) Prior on the instantaneous transition rate q_{01} set to U(0, 0.2). Numbers on Nodes correspond to Supplementary Table 25. (b) Prior on the instantaneous transition rate q_{01} set to U(0, 20). Numbers on Nodes correspond to Supplementary Table 15. (c) Prior on the instantaneous transition rate q_{01} set to U(0, 200). Numbers on Nodes correspond to Supplementary Table 20. Colors are as shown in Figure 1 in the main manuscript. Pie charts represent the mean posterior probabilities from MCMC run using 10,000 trees sampled from the posterior distribution of divergence time estimation analyses.

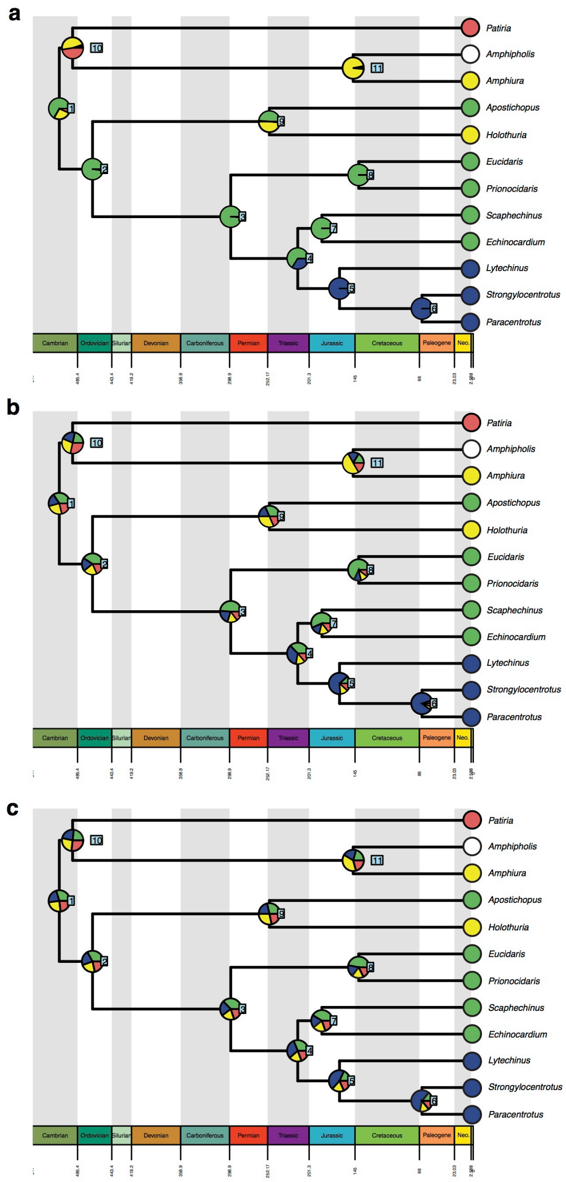

Supplementary Figure 10. Ancestral state reconstruction of *tbrain* gene expression patterns using a single-rate Markov model with differing instantaneous transition rates. (a) Prior on the instantaneous transition rate q_{01} set to U(0, 0.2). Numbers on Nodes correspond to Supplementary Table 24. (b) Prior on the instantaneous transition rate q_{01} set to U(0, 20). Numbers on Nodes correspond to Supplementary Table 14 (c) Prior on the instantaneous transition rate q_{01} set to U(0, 200). Numbers on Nodes correspond to Supplementary Table 19. Colors are as shown in Figure 1 in the main manuscript. Pie charts represent the mean posterior probabilities from MCMC run using 10,000 trees sampled from the posterior distribution of divergence time estimation analyses.

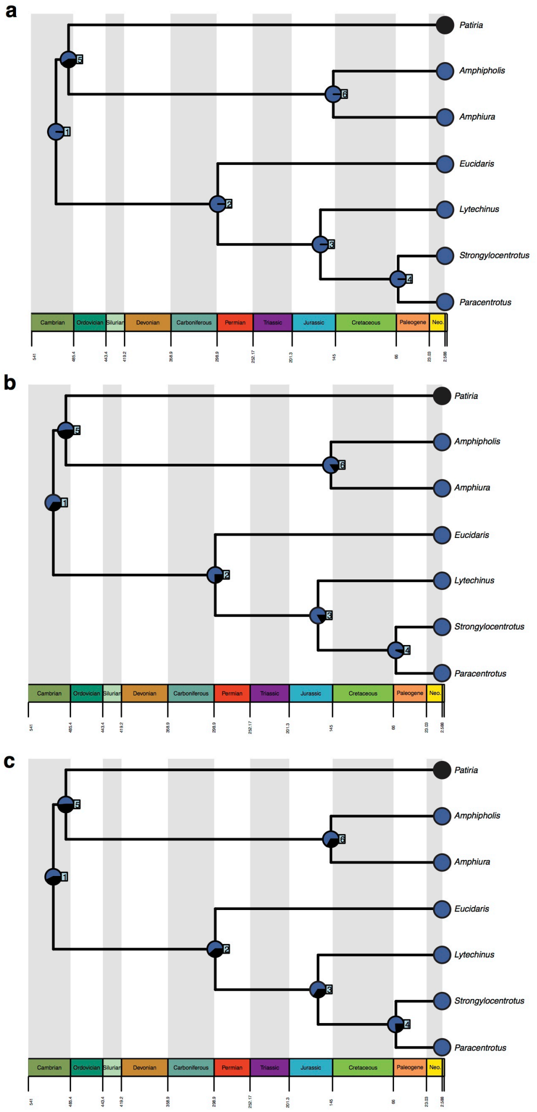

Supplementary Figure 11. Ancestral state reconstruction of *vegfr* gene expression patterns using a single-rate Markov model with differing priors on instantaneous transition rates. (a) Prior on the instantaneous transition rate q_{01} set to U(0, 0.2). Numbers on Nodes correspond to Supplementary Table 26. (b) Prior on the instantaneous transition rate q_{01} set to U(0, 20). Numbers on Nodes correspond to Supplementary Table 16. (c) Prior on the instantaneous transition rate q_{01} set to U(0, 200). Numbers on Nodes correspond to Supplementary Table 21. q_{01} set to U(0, 20). Colors are as shown in Figure 1 in the main manuscript. Pie charts represent the mean posterior probabilities from MCMC run using 10,000 trees sampled from the posterior distribution of divergence time estimation analyses.

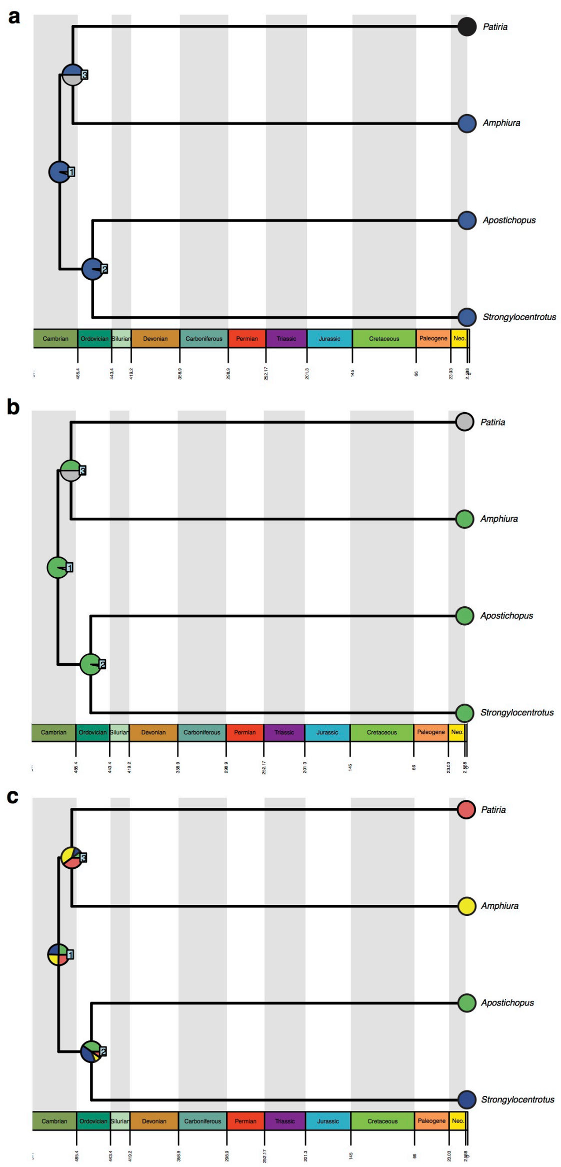

Supplementary Figure 12. Ancestral state reconstruction of *alx1, ets1, and tbrain* gene expression patterns using a single-rate Markov model with tree tips pruned to include only one taxon per class. (a) *alx1.* Numbers on Nodes correspond to Supplementary Table 28. (b) *ets1.* Numbers on Nodes correspond to Supplementary Table 29. (c) *tbrain.* Numbers on Nodes correspond to Supplementary Table 30. Colors are as shown in Figure 1 in the main manuscript. Pie charts represent the mean posterior probabilities from MCMC run using 10,000 trees sampled from the posterior distribution of divergence time estimation analyses.

Supplementary Tables

Supplementary Table 1. Log Bayes Factors showing support for single-rate or multi-rate models for ancestral state reconstructions of *alx1*, *erg*, *ets1*, *tbrain,* and *vegfr*. Interpreted according to Kass and Raftery [4], 2*Log Bayes Factors which are less than 2 indicate support that "is not worth more than a bare mention".

Gene	2*Log Bayes Factor
alx1	0.01
erg	0.002
ets1	0.01
vegfr	0.04
tbrain	-0.02

Supplementary Table 2. Ancestral state reconstructions of gene expression patterns of *alx1* using a single rate model. This is the same as shown in Figure 2 in the main manuscript. Node values correspond to nodes in Supplementary Figure 3a.

Node	Expressed in SM	Mesoderm/No SM
1	0.97	0.03
2	0.98	0.02
3	0.99	0.01
4	1.00	0.00
5	0.99	0.01
6	1.00	0.00
7	0.99	0.01
8	0.99	0.01
9	0.98	0.02
10	0.58	0.42
11	0.99	0.01

Supplementary Table 3. Ancestral state reconstructions of gene expression patterns of *ets1* using a single rate model. This is the same as shown in Figure 2 in the main manuscript. Node values correspond to nodes in Supplementary Figure 4a.

Node	Expressed in NSM and SM	Mesoderm/No SM
1	0.97	0.03
2	0.98	0.02
3	0.99	0.01
4	1.00	0.00
5	0.99	0.01
6	1.00	0.00
7	0.99	0.01
8	0.99	0.01
9	0.98	0.02
10	0.58	0.42
11	0.99	0.01

Supplementary Table 4. Ancestral state reconstructions of gene expression patterns of *tbrain* using a single rate model. This is the same as shown in Figure 2 in the main manuscript. Node values correspond to nodes in Supplementary Figure 6a.

Node	Expressed in NSM and SM	Expressed in SM	Expressed in NSM, SM and Endoderm	Expressed in Endoderm, Mesoderm, and no early SM lineage
1	0.51	0.10	0.24	0.15
2	0.69	0.10	0.13	0.07
3	0.83	0.12	0.03	0.03
4	0.53	0.42	0.03	0.03
5	0.01	0.96	0.01	0.01
6	0.00	0.99	0.00	0.00
7	0.92	0.03	0.03	0.03
8	0.96	0.01	0.01	0.01
9	0.41	0.09	0.41	0.09
10	0.15	0.15	0.35	0.35
11	0.09	0.09	0.74	0.09

Supplementary Table 5. Ancestral state reconstructions of gene expression patterns of *erg* using a single rate model. This is the same as shown in Figure 3 in the main manuscript. Node values correspond to nodes in Supplementary Figure 5a.

Node	Expressed in NSM	Expressed in mesoderm, and no early SM lineage
1	0.960	0.040
2	0.977	0.023
3	0.988	0.012
4	0.993	0.007
5	0.500	0.500

Supplementary Table 6. Ancestral state reconstructions of gene expression patterns of *vegfr* using a single rate model. This is the same as shown in Figure 3 in the main manuscript. Node values correspond to nodes in Supplementary Figure 2a.

Node	Expressed in SM	Not expressed/No SM
1	0.90	0.10
2	0.98	0.02
3	0.99	0.01
4	1.00	0.00
5	0.57	0.43
6	0.99	0.01

Supplementary Table 7. Ancestral state reconstructions of gene expression patterns of *alx1* using a two rate model. Node values correspond to nodes in Supplementary Figure 3b.

Node	Expressed in SM	Mesoderm/No SM
1	0.929547154	0.070452846
2	0.975981664	0.024018336
3	0.991809581	0.00819042
4	0.997764378	0.002235622
5	0.990403118	0.009596882
6	0.997273202	0.002726798
7	0.982724199	0.017275801
8	0.988688367	0.011311633
9	0.970361718	0.029638282
10	0.539445143	0.460554857
11	0.987626729	0.012373271

Supplementary Table 8. Ancestral state reconstructions of gene expression patterns of *ets1* using a two-rate model. Node values correspond to nodes in Supplementary Figure 4b.

Node	Expressed in NSM and SM	Mesoderm/No SM
1	0.928000206	0.071999794
2	0.975206882	0.024793118
3	0.991518104	0.008481896
4	0.997688264	0.002311736
5	0.990073671	0.009926329
6	0.997140457	0.002859543
7	0.982240293	0.017759707
8	0.988473073	0.011526927
9	0.969549469	0.030450531
10	0.539739797	0.460260203
11	0.987493189	0.012506811

Supplementary Table 9. Ancestral state reconstructions of gene expression patterns of *tbrain* using a multi-rate model. Node values correspond to nodes in Supplementary Figure 6b.

Node	Expressed in NSM and SM	Expressed in SM	Expressed in NSM, SM and Endoderm	Expressed in Endoderm, Mesoderm, and no early SM lineage
1	0.48	0.09	0.28	0.15
2	0.70	0.10	0.14	0.06
3	0.83	0.13	0.02	0.02
4	0.54	0.40	0.03	0.02
5	0.01	0.97	0.01	0.01
6	0.00	0.99	0.00	0.00
7	0.94	0.02	0.02	0.02
8	0.96	0.01	0.01	0.01
9	0.42	0.08	0.42	0.08
10	0.13	0.13	0.36	0.38
11	0.08	0.07	0.77	0.08

Supplementary Table 10. Ancestral state reconstructions of gene expression patterns of *erg* using a two-rate model. Node values correspond to nodes in Supplementary Figure 5b.

Node	Expressed in NSM	Expressed in mesoderm, and no early SM lineage
1	0.947	0.053
2	0.981	0.019
3	0.990	0.010
4	0.994	0.006
5	0.537	0.463

Supplementary Table 11. Ancestral state reconstructions of gene expression patterns of *vegfr* using a two-rate model. Node values correspond to nodes in Supplementary Figure 2b.

Node	Expressed in SM	Not expressed/No SM
1	0.87	0.13
2	0.98	0.02
3	0.99	0.01
4	1.00	0.00
5	0.58	0.42
6	0.99	0.01

Supplementary Table 12. Ancestral state reconstructions of gene expression patterns of *alx1* using a single rate model with the prior on the instantaneous transition rate q_{01} set to U(0, 20). Node values correspond to nodes in Supplementary Figure 7b.

Node	Expressed in SM	Mesoderm/No SM
1	0.89	0.11
2	0.90	0.10
3	0.94	0.06
4	0.96	0.04
5	0.95	0.05
6	0.98	0.02
7	0.93	0.07
8	0.95	0.05
9	0.90	0.10
10	0.56	0.44
11	0.95	0.05

Supplementary Table 13. Ancestral state reconstructions of gene expression patterns of *ets1* using a single rate model with the prior on the instantaneous transition rate q_{01} set to U(0, 20). Node values correspond to nodes in Supplementary Figure 8b.

Node	Expressed in NSM and SM	Mesoderm/No SM
1	0.89	0.11
2	0.90	0.10
3	0.94	0.06
4	0.96	0.04
5	0.95	0.05
6	0.98	0.02
7	0.93	0.07
8	0.95	0.05
9	0.90	0.10
10	0.56	0.44
11	0.95	0.05

Supplementary Table 14. Ancestral state reconstructions of gene expression patterns of *tbrain* using a single rate model with the prior on the instantaneous transition rate q_{01} set to U(0, 20). Node values correspond to nodes in Supplementary Figure 10b.

Node	Expressed in NSM and SM	Expressed in SM	Expressed in NSM, SM and Endoderm	Expressed in Endoderm, Mesoderm, and no early SM lineage
1	0.34	0.20	0.25	0.21
2	0.41	0.20	0.21	0.18
3	0.49	0.21	0.15	0.15
4	0.37	0.35	0.14	0.14
5	0.12	0.65	0.12	0.12
6	0.05	0.85	0.05	0.05
7	0.57	0.14	0.14	0.14
8	0.68	0.11	0.11	0.11
9	0.32	0.18	0.32	0.18
10	0.22	0.22	0.28	0.28
11	0.17	0.17	0.50	0.17

Supplementary Table 15. Ancestral state reconstructions of gene expression patterns of *erg* using a single rate model with the prior on the instantaneous transition rate q_{01} set to U(0, 20). Node values correspond to nodes in Supplementary Figure 9b.

Node	Expressed in NSM	Expressed in mesoderm, and no early SM lineage
1	0.675	0.325
2	0.693	0.307
3	0.749	0.251
4	0.823	0.177
5	0.500	0.500

Supplementary Table 16. Ancestral state reconstructions of gene expression patterns of *vegfr* using a single rate model with the prior on the instantaneous transition rate q_{01} set to U(0, 20). Node values correspond to nodes in Supplementary Figure 11b.

Node	Expressed in SM	Not expressed/No SM
1	0.66	0.34
2	0.75	0.25
3	0.84	0.16
4	0.94	0.06
5	0.53	0.47
6	0.85	0.15

Supplementary Table 17. Ancestral state reconstructions of gene expression patterns of *alx1* using a single rate model with the prior on the instantaneous transition rate q_{01} set to U(0, 200). Node values correspond to nodes in Supplementary Figure 7c.

Node	Expressed in SM	Mesoderm/No SM
1	0.81	0.19
2	0.82	0.18
3	0.85	0.15
4	0.86	0.14
5	0.86	0.14
6	0.90	0.10
7	0.84	0.16
8	0.86	0.14
9	0.82	0.18
10	0.55	0.45
11	0.86	0.14

Supplementary Table 18. Ancestral state reconstructions of gene expression patterns of *ets1* using a single rate model with the prior on the instantaneous transition rate q_{01} set to U(0, 200). Node values correspond to nodes in Supplementary Figure 8c

Node	Expressed in NSM and SM	Mesoderm/No SM
1	0.81	0.19
2	0.82	0.18
3	0.85	0.15
4	0.86	0.14
5	0.86	0.14
6	0.90	0.10
7	0.84	0.16
8	0.86	0.14
9	0.82	0.18
10	0.55	0.45
11	0.86	0.14

Supplementary Table 19. Ancestral state reconstructions of gene expression patterns of *tbrain* using a single rate model with the prior on the instantaneous transition rate q_{01} set to U(0, 200). Node values correspond to nodes in Supplementary Figure 10c.

Node	Expressed in NSM and SM	Expressed in SM	Expressed in NSM, SM and Endoderm	Expressed in Endoderm, Mesoderm, and no early SM lineage
1	0.30	0.22	0.25	0.23
2	0.33	0.23	0.23	0.22
3	0.37	0.23	0.20	0.20
4	0.31	0.30	0.19	0.19
5	0.18	0.45	0.18	0.18
6	0.14	0.58	0.14	0.14
7	0.41	0.20	0.20	0.20
8	0.47	0.18	0.18	0.18
9	0.28	0.22	0.28	0.22
10	0.23	0.23	0.27	0.27
11	0.21	0.21	0.38	0.21

Supplementary Table 20. Ancestral state reconstructions of gene expression patterns of *erg* using a single rate model with the prior on the instantaneous transition rate q_{01} set to U(0, 200). Node values correspond to nodes in Supplementary Figure 9c.

Node	Expressed in NSM	Expressed in mesoderm, and no early SM lineage
1	0.560	0.440
2	0.566	0.434
3	0.586	0.414
4	0.619	0.381
5	0.500	0.500

Supplementary Table 21. Ancestral state reconstructions of gene expression patterns of *vegfr* using a single rate model with the prior on the instantaneous transition rate q_{01} set to U(0, 200). Node values correspond to nodes in Supplementary Figure 11c.

Node	Expressed in SM	Not expressed/No SM
1	0.57	0.43
2	0.61	0.39
3	0.66	0.34
4	0.76	0.24
5	0.51	0.49
6	0.67	0.33

Supplementary Table 22. Ancestral state reconstructions of gene expression patterns of *alx1* using a single rate model with the prior on the instantaneous transition rate q_{01} set to U(0, 0.2). Node values correspond to nodes in Supplementary Figure 7a.

Node	Expressed in SM	Mesoderm/No SM
1	1.00	0.00
2	1.00	0.00
3	1.00	0.00
4	1.00	0.00
5	1.00	0.00
6	1.00	0.00
7	1.00	0.00
8	1.00	0.00
9	1.00	0.00
10	0.59	0.41
11	1.00	0.00

Supplementary Table 23. Ancestral state reconstructions of gene expression patterns of *ets1* using a single rate model with the prior on the instantaneous transition rate q_{01} set to U(0, 0.2). Node values correspond to nodes in Supplementary Figure 8a.

Node	Expressed in NSM and SM	Mesoderm/No SM
1	1.00	0.00
2	1.00	0.00
3	1.00	0.00
4	1.00	0.00
5	1.00	0.00
6	1.00	0.00
7	1.00	0.00
8	1.00	0.00
9	1.00	0.00
10	0.59	0.41
11	1.00	0.00

Supplementary Table 24. Ancestral state reconstructions of gene expression patterns of *tbrain* using a single rate model with the prior on the instantaneous transition rate q_{01} set to U(0, 0.2). Node values correspond to nodes in Supplementary Figure 10a.

Node	Expressed in NSM and SM	Expressed in SM	Expressed in NSM, SM and Endoderm	Expressed in Endoderm, Mesoderm, and no early SM lineage
1	0.67	0.00	0.26	0.07
2	0.98	0.00	0.02	0.00
3	0.99	0.01	0.00	0.00
4	0.66	0.34	0.00	0.00
5	0.00	1.00	0.00	0.00
6	0.00	1.00	0.00	0.00
7	1.00	0.00	0.00	0.00
8	1.00	0.00	0.00	0.00
9	0.49	0.01	0.49	0.01
10	0.03	0.03	0.47	0.47
11	0.01	0.01	0.96	0.01

Supplementary Table 25. Ancestral state reconstructions of gene expression patterns of *erg* using a single rate model with the prior on the instantaneous transition rate q_{01} set to U(0, 0.2). Node values correspond to nodes in Supplementary Figure 9a.

Node	Expressed in NSM	Expressed in mesoderm, and no early SM lineage
1	0.9975	0.0025
2	0.9997	0.0003
3	0.9999	0.0001
4	0.9999	0.0001
5	0.5000	0.5000

Supplementary Table 26. Ancestral state reconstructions of gene expression patterns of *vegfr* using a single rate model with the prior on the instantaneous transition rate q_{01} set to U(0, 0.2). Node values correspond to nodes in Supplementary Figure 11a.

Node	Expressed in SM	Not expressed/No SM
1	0.99	0.01
2	1.00	0.00
3	1.00	0.00
4	1.00	0.00
5	0.59	0.41
6	1.00	0.00

Supplementary Table 27. Bayes Factor hypothesis tests comparing support for different ancestral states at the MRCA of asterozoans (Node 10 for *alx1, erg,* and *ets1* ; Node 5 for *vegfr*). Interpretations are based on the table in Kass and Raftery [4] where 2*Log Bayes Factors between six and ten indicates "strong" support for a given model. In this case, those are models where the asterozoan MRCA is fixed as expression in the skeletogenic mesenchyme for *alx1* and *vegfr* and expression in the skeletogenic mesenchyme and mesoderm for *ets1*.

	2*Log Bayes Factor
alx1	7.92
erg	7.76
ets1	7.91
vegfr	5.67

Supplementary Table 28. Ancestral state reconstructions of gene expression patterns of *alx1* using a single rate model and using a sample from the prior where trees were pruned to include only one representative taxon per class. Node values correspond to nodes in Supplementary Figure 12a.

Node	Expressed in SM	Mesoderm/No SM
1	0.95	0.05
2	0.97	0.03
3	0.50	0.50

Supplementary Table 29. Ancestral state reconstructions of gene expression patterns of *ets1* using a single rate model and using a sample from the prior where trees were pruned to include only one representative taxon per class. Node values correspond to nodes in Supplementary Figure 12b.

Node	Expressed in NSM and SM	Mesoderm/No SM
1	0.95	0.05
2	0.97	0.03
3	0.50	0.50

Supplementary Table 30. Ancestral state reconstructions of gene expression patterns of *tbrain* using a single rate model and using a sample from the prior where trees were pruned to include only one representative taxon per class. Node values correspond to nodes in Supplementary Figure 12c.

Node	Expressed in NSM and SM	Expressed in SM	Expressed in NSM, SM and Endoderm	Expressed in Endoderm, Mesoderm, and no early SM lineage
1	0.25	0.25	0.25	0.25
2	0.40	0.40	0.10	0.10
3	0.10	0.10	0.40	0.40

Supplementary References

1 Telford, M. J. *et al.* Phylogenomic analysis of echinoderm class relationships supports Asterozoa. *Proceedings of the Royal Society B: Biological Sciences* **281**, 20140479 (2014).

2 Smith, A. B. *et al.* Testing the molecular clock: molecular and paleontological estimates of divergence times in the Echinoidea (Echinodermata). *Mol Biol Evol* **23**, 1832-1851, doi:10.1093/molbev/msl039 (2006).

3 Mongiardino Koch, N. *et al.* A phylogenomic resolution of the sea urchin tree of life. *BMC Evol Biol* **18**, 189, doi:10.1186/s12862-018-1300-4 (2018).

4 Kass, R. E. & Raftery, A. E. Bayes Factors. *Journal of the American Statistical Association* **90**, 773-795 (1995).
