# Supplementary material for: Cell type phylogenetics informs the evolutionary origin of echinoderm larval skeletogenic cell identity
Source: Commun Biol. 2019 May 3;2:160. doi: 10.1038/s42003-019-0417-3 (PMC6499829; doi:10.1038/s42003-019-0417-3)
Supplement: Supplementary file 4 — Reporting Summary [file 42003_2019_417_MOESM4_ESM.pdf]

nature research

Corresponding author(s): Erkenbrack and Thompson

Reporting Summary

Nature Research wishes to improve the reproducibility of the work that we publish. This form provides structure for consistency and transparency in reporting. For further information on Nature Research policies, see Authors & Referees and the Editorial Policy Checklist.

Statistical parameters

When statistical analyses are reported, confirm that the following items are present in the relevant location (e.g. figure legend, table legend, main text, or Methods section).

n/a	Confirmed	
☒	☐	The exact sample size (n) for each experimental group/condition, given as a discrete number and unit of measurement
☒	☐	An indication of whether measurements were taken from distinct samples or whether the same sample was measured repeatedly
☒	☐	The statistical test(s) used AND whether they are one- or two-sided<br>*Only common tests should be described solely by name; describe more complex techniques in the Methods section.*
☒	☐	A description of all covariates tested
☒	☐	A description of any assumptions or corrections, such as tests of normality and adjustment for multiple comparisons
☒	☐	A full description of the statistics including central tendency (e.g. means) or other basic estimates (e.g. regression coefficient) AND variation (e.g. standard deviation) or associated estimates of uncertainty (e.g. confidence intervals)
☒	☐	For null hypothesis testing, the test statistic (e.g. F, t, r) with confidence intervals, effect sizes, degrees of freedom and P value noted<br>*Give P values as exact values whenever suitable.*
☐	☒	For Bayesian analysis, information on the choice of priors and Markov chain Monte Carlo settings
☒	☐	For hierarchical and complex designs, identification of the appropriate level for tests and full reporting of outcomes
☒	☐	Estimates of effect sizes (e.g. Cohen's d, Pearson's r), indicating how they were calculated
☒	☐	Clearly defined error bars<br>*State explicitly what error bars represent (e.g. SD, SE, CI)*

Our web collection on statistics for biologists may be useful.

Software and code

Policy information about availability of computer code

Data collection	All data were complied from previously published literature, as mentioned in the supplementary information file. We have also included all code and data files necessary to repeat all analyses.
Data analysis	All analyses were performed using custom code or by using previously published analytical programs. We've included all data files and code necessary to repeat our analyses.

For manuscripts utilizing custom algorithms or software that are central to the research but not yet described in published literature, software must be made available to editors/reviewers upon request. We strongly encourage code deposition in a community repository (e.g. GitHub). See the Nature Research guidelines for submitting code & software for further information.

Data

Policy information about availability of data

All manuscripts must include a data availability statement. This statement should provide the following information, where applicable:

- Accession codes, unique identifiers, or web links for publicly available datasets
- A list of figures that have associated raw data
- A description of any restrictions on data availability

All data and code necessary to repeat analyses is available on GitHub at https://github.com/jthechino/Erkenbrack_-_Thompson

Field-specific reporting

Please select the best fit for your research. If you are not sure, read the appropriate sections before making your selection.

☐ Life sciences ☐ Behavioural & social sciences ☒ Ecological, evolutionary & environmental sciences

For a reference copy of the document with all sections, see nature.com/authors/policies/ReportingSummary-flat.pdf

Ecological, evolutionary & environmental sciences study design

All studies must disclose on these points even when the disclosure is negative.

Study description	We used a Markov Model to analyze the evolution of gene expression in embryonic echinoderms. Analyses were run in a Bayesian Framework.
Research sample	We attempted to include all indirect-developing echinoderm species where gene sequence and in situ hybridization data were available, as described in the supplementary methods. These data were complied from previously published literature. A table detailing the source of all data is included in the supplementary information.
Sampling strategy	No sample size calculation was performed, though we attempted to include all indirect-developing echinoderm species where gene sequence and in situ hybridization data were available, as described in the supplementary methods.
Data collection	Data were collected from previously published literature by lead author Eric Erkenbrack as described in the main text and supplementary file.
Timing and spatial scale	All data are from extant echinoderm species.
Data exclusions	No data were excluded.
Reproducibility	Analyses were run multiple times and with different prior and model settings. We have included all code and data files necessary for others to repeat our analyses.
Randomization	Randomization was not appropriate for this study, though we did use Bayesian analyses to explicitly integrate over uncertainty associated with our analyses.
Blinding	Blinding was not necessary for this study, though we utilized sensitivity analyses to check the sensitivity of our results to prior and model choice.

Did the study involve field work? ☐ Yes ☒ No

Reporting for specific materials, systems and methods

Materials & experimental systems

n/a	Involved in the study
☒	☐ Unique biological materials
☒	☐ Antibodies
☒	☐ Eukaryotic cell lines
☐	☒ Palaeontology
☒	☐ Animals and other organisms
☒	☐ Human research participants

Methods

n/a	Involved in the study
☒	☐ ChIP-seq
☒	☐ Flow cytometry
☒	☐ MRI-based neuroimaging

Palaeontology

Specimen provenance	No specimens were explicitly described in this paper, we used specimens from previously published literature to time-calibrate our phylogeny. These specimens were cited appropriately.
Specimen deposition	We did not collect any new specimens.

Dating methods

No new dates were provided, though the age of specimens we used for our analyses is detailed in the supplementary file.

☒ Tick this box to confirm that the raw and calibrated dates are available in the paper or in Supplementary Information.
